# Crystal structures of four organic salts of trihexyphenidyl at 90 K

**DOI:** 10.1107/S2056989023005960

**Published:** 2023-07-14

**Authors:** Vinaya, Yeriyur B. Basavaraju, Hemmige S. Yathirajan, Sean Parkin

**Affiliations:** a Department of Studies in Chemistry, University of Mysore, Manasagangotri, Mysuru-570 006, India; b Department of Chemistry, University of Kentucky, Lexington, KY, 40506-0055, USA; Katholieke Universiteit Leuven, Belgium

**Keywords:** trihexyphenid­yl, trihexyphenidylium cation, crystal structure, disorder, twinning, non-merohedry, hydrogen bonding

## Abstract

The syntheses and crystal structure studies of four organic salts of trihexyphenidyl, *viz*., trihexyphenidylium [1-(3-cyclo­hexyl-3-hy­droxy-3-phenyl­prop­yl)piperidin-1-ium] 4-nitro­benzoate, C_20_H_32_NO^+^·C_7_H_4_NO_4_^−^ (**I**), trihexyphenidylium 4-hy­droxy­benzoate, C_20_H_32_NO^+^·C_7_H_5_O^−^ (**II**), trihexyphenidylium 4-bromo­benzoate, C_20_H_32_NO^+^·C_7_H_4_BrO_2_^−^ (**III**), and trihexyphenidylium thio­phene-2-carboxyl­ate hemihydrate, 2C_20_H_32_NO^+^·2C_5_H_3_O_2_S^−^·H_2_O (**IV**), con­ducted at 90 K are described. Structures **I**, **II**, and **III** are solvent free with one cation–anion pair per asymmetric unit, while **IV** crystallizes as a hemihydrate, having two cation–anion pairs and one water of crystallization in its asymmetric unit. Structures **I** and **III** exhibit configurational disorder of the cation. Structure **IV** also exhibits disorder, but only of the thio­phene-2-carboxyl­ate anions. Structure **II** is a non-merohedric twin by a twofold rotation about [403]. The main supra­molecular motifs in **I**, **II**, and **III** are similar *R* ^2^_2_(10) rings between cation–anion pairs, although their packing within the crystals is distinct. As a consequence of having two cation–anion pairs and a water mol­ecule in its asymmetric unit, the packing in **IV** is by far the most complex of the four structures, its hydrogen-bonding patterns being quite different from **I**, **II**, or **III**. In all the crystals studied, N—H⋯O, O—H⋯O, and C—H⋯O inter­actions are observed, plus C—H⋯Br close contacts for **III**.

## Abstract 2

The low-temperature crystal structures of four organic salts of the anti-spasmodic drug trihexyphenidyl are presented.

## 1. Chemical context

Trihexyphenidyl, systematic name 1-cyclo­hexyl-1-phenyl-3-(piperidin-1-yl)propan-1-ol, is an anti­spasmodic drug used to treat stiffness, tremors, spasms, and poor muscle control. It can be used in the treatment of psychotic depression (Roth *et al.*, 1994 ▸; Seeman & Tallerico, 1998 ▸; Silvestre & Prous, 2005 ▸). In addition, trihexyphenidyl is well established as a treatment for symptomatic relief in cases of Parkinson’s disease (Doshay *et al.*, 1954 ▸). Trihexyphenidyl contains a chiral carbon atom, although medicinal formulations are racemates. It is generally administered as the hydro­chloride salt, the structure of which was published by Maccaroni *et al.* (2010 ▸), although structures have also been reported for neutral trihexyphenidyl (Camerman & Camerman, 1972 ▸), and the trihexyphenidylium 3,5-di­nitro­benzoate salt (Shaibah *et al.*, 2019 ▸).

In view of the medicinal importance of trihexyphenidyl, this paper reports the crystal structures of some salts of trihexyphenidyl with organic acids, *viz.*, trihexyphenidylium 4-nitro­benzoate, C_26_H_36_NO_3_ (**I**), trihexyphenidylium 4-hy­droxy­benzoate, C_27_H_37_NO_4_ (**II**), trihexyphenidylium 4-bromo­benzoate, C_27_H_36_NO_3_Br (**III**) and trihexyphenidylium thio­phene-2-carboxyl­ate, which crystallizes as a hemihydrate, C_25_H_35_NO_3_S·0.5H_2_O (**IV**). 
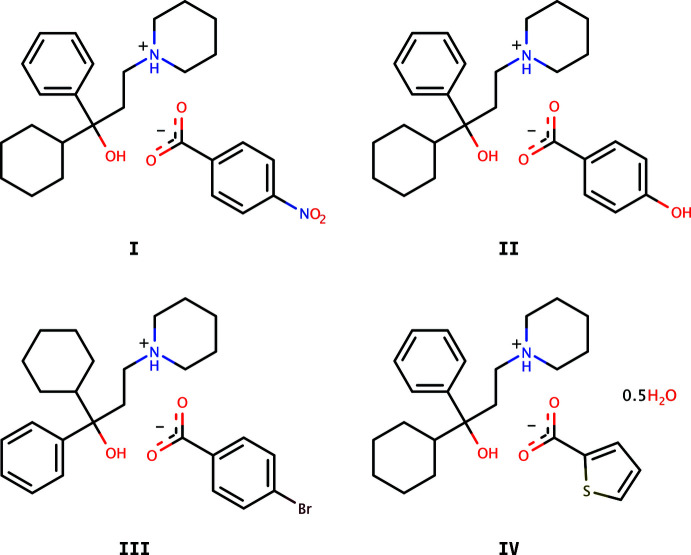


## 2. Structural commentary

**Figure 1:**
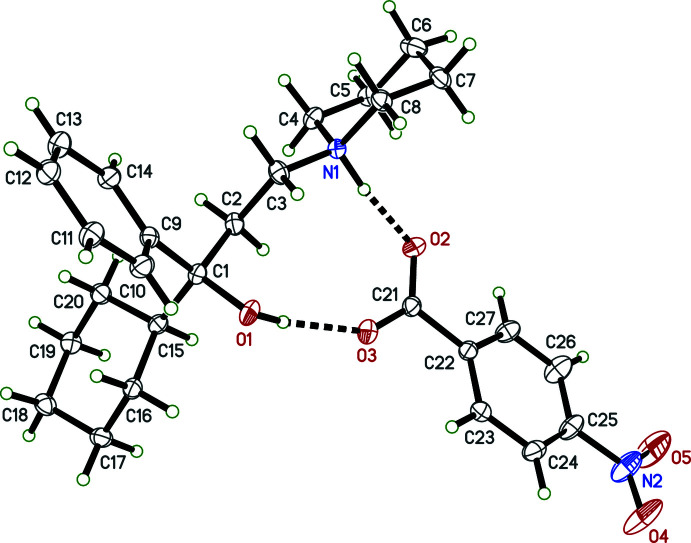
An ellipsoid plot (50%) probability of **I**. Hydrogen bonds are shown as dashed lines. To enhance clarity, only one component of disorder for the cation is shown.

Individual neutral trihexyphenidyl mol­ecules contain a chiral carbon atom. In structures **I**, **II**, **III**, **IV**, each trihexyphenidylium cation also includes a chiral carbon, with atoms C1 (C1*A* and C1*B* in **IV**) being the stereogenic centre. Nevertheless, medicinal formulations are racemic, and all four crystal structures (Figs. 1 ▸–4 ▸ ▸ ▸) determined here are centrosymmetric and therefore also strictly racemic. Structures **I**, **II**, and **III** are solvent free with one cation–anion pair per asymmetric unit, while **IV** crystallized as a hemihydrate, having two cation–anion pairs and one water of crystallization in its asymmetric unit.

Structures **I** and **III** exhibit configurational disorder (see *e.g.* Parkin *et al.*, 2023 ▸; Vinaya *et al.*, 2023 ▸) of the cation. This disorder switches the positions and overlays the phenyl and cyclo­hexyl rings, thereby superimposing *R* and *S* isomers in roughly equal refined proportions [0.503 (4):0.497 (4) in **I** and 0.508 (5):0.492 (5) in **III**], as shown for **I** in Fig. 5 ▸. Structure **IV** also exhibits disorder, but of the thio­phene-2-carboxyl­ate anions [major:minor fractions are 0.795 (2):0.805 (2) and 0.953 (2):0.047 (2) for the inequivalent anion sites]. Structure **II** is a mon-merohedric twin (see *e.g.* Sevvana *et al.*, 2019 ▸; Parkin, 2021 ▸) by a twofold rotation about [403], with similar twin-component fractions [0.5298 (9) and 0.4702 (9)]. The treatment of disorder and twinning are described in more detail in section 6 (*Refinement*).

The conformations of the trihexyphenidylium cations are determined, in large part, by torsion angles about the C1—C2, C2—C3, C3—N1, C1—C9, and C1—C15 bonds. These are qu­anti­fied in Table 1 ▸, although for ease of comparison, the variability in cation conformations is better illustrated by an overlay plot, shown in Fig. 6 ▸.

## 3. Supra­molecular features

The main supra­molecular motifs in **I**, **II**, and **III** are 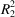 (10) hydrogen-bonded rings involving N—H and O—H donors from the cations and the carboxyl­ate group of their respective anions. These ring structures are shown in the ellipsoid plots (Figs. 1 ▸–3 ▸ ▸), while Figs. 7 ▸–9 ▸ ▸ show how they pack within their unit cells.

For **I** there are no other strong inter­molecular inter­actions, though there is a weaker C4—H4*B*⋯O1^i^ [symmetry code: (i) *x* − 1, *y*, *z*] contact between mol­ecules adjacent along the *a*-axis direction. See Table 2 ▸ for details.

In **II**, the 4-hy­droxy group of the anion is also involved in hydrogen bonding (Fig. 8 ▸). Atom O3 of the carboxyl­ate acts as a bifurcated acceptor for the N1—H1*N*⋯O3 hydrogen bond within the 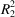 (10) ring and for an O4^ii^—H4*O* ^ii^⋯O3 [symmetry code: (ii) *x*, −*y*, *z* − 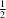 ] hydrogen bond. There are also a few weaker C—H⋯O inter­actions. All these inter­actions are qu­anti­fied in Table 3 ▸.

In **III**, in addition to the aforementioned ring motif, there are short contacts between C3—H3*A* and Br1 of a screw-related (−*x* + 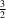 , *y* + 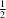 , −*z* + 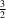 ) anion and between C4—H4*A* and O3 of a translation-related (*x* − 1, *y*, *z*) anion. Full details are given in Table 4 ▸. A view of the packing is shown in Fig. 9 ▸.

**Figure 4:**
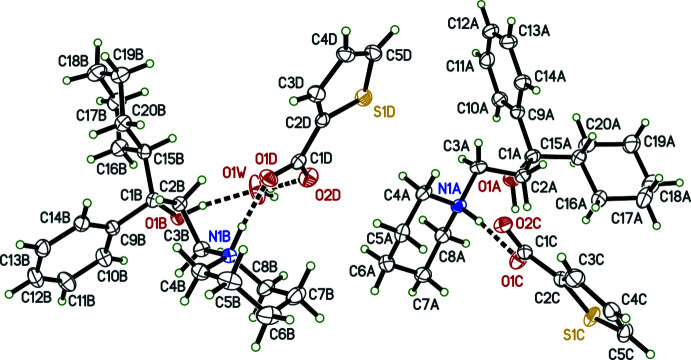
An ellipsoid plot (50%) probability of **IV**. Hydrogen bonds are shown as dashed lines. To enhance clarity, only one component of disorder for the anions is shown.

**Figure 5:**
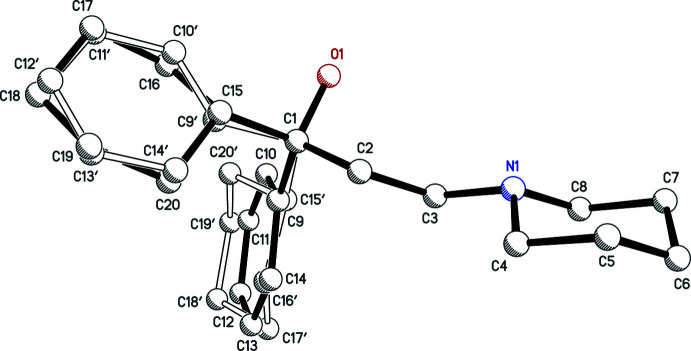
Configurational disorder of the trihexyphenidylium cation in **I** showing the superposition of phenyl and cyclo­hexyl rings. The disorder in **III** is similar. Hydrogen atoms are omitted.

**Figure 6:**
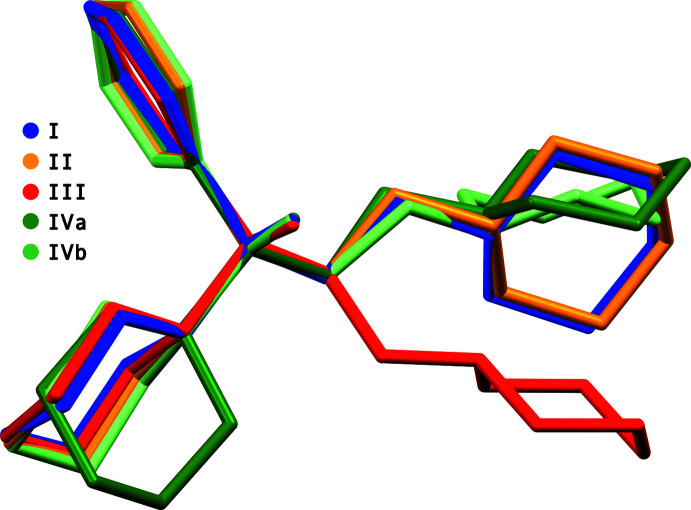
An overlay of five independent trihexyphenidylium cations from structures **I**, **II**, **III**, and **IV**, showing the conformational variability.

**Figure 7:**
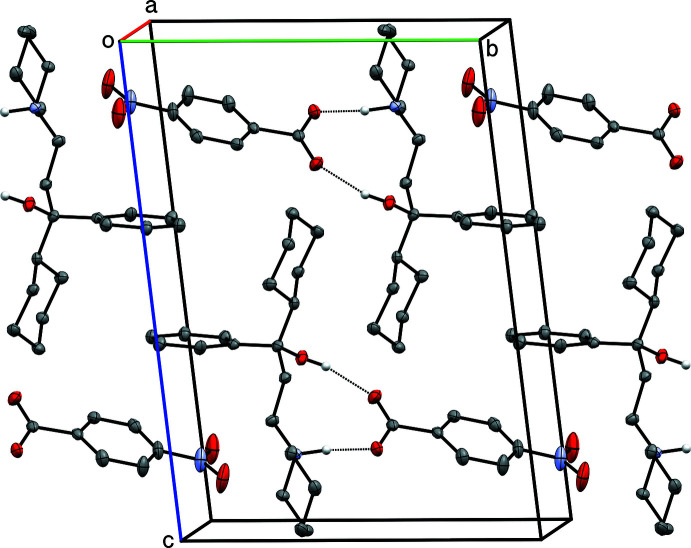
A partial packing plot of **I**, viewed approximately down the *a*-axis. Hydrogen bonds are drawn as dotted lines. Hydrogen atoms not involved in hydrogen bonds are omitted.

**Figure 8:**
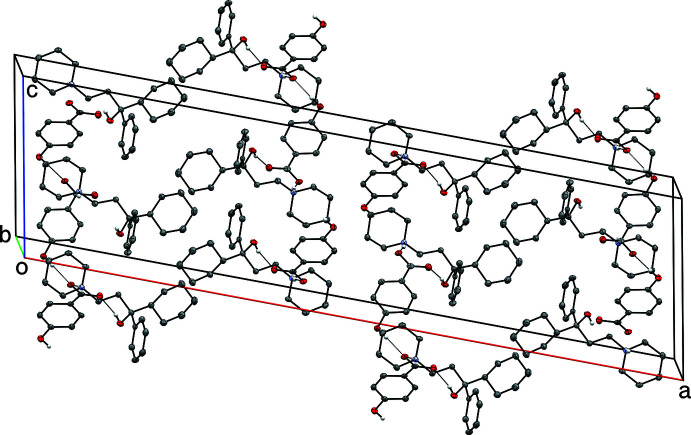
A partial packing plot of **II**, viewed approximately down the *b*-axis. Hydrogen bonds are drawn as dotted lines. Hydrogen atoms not involved in hydrogen bonds are omitted.

**Figure 9:**
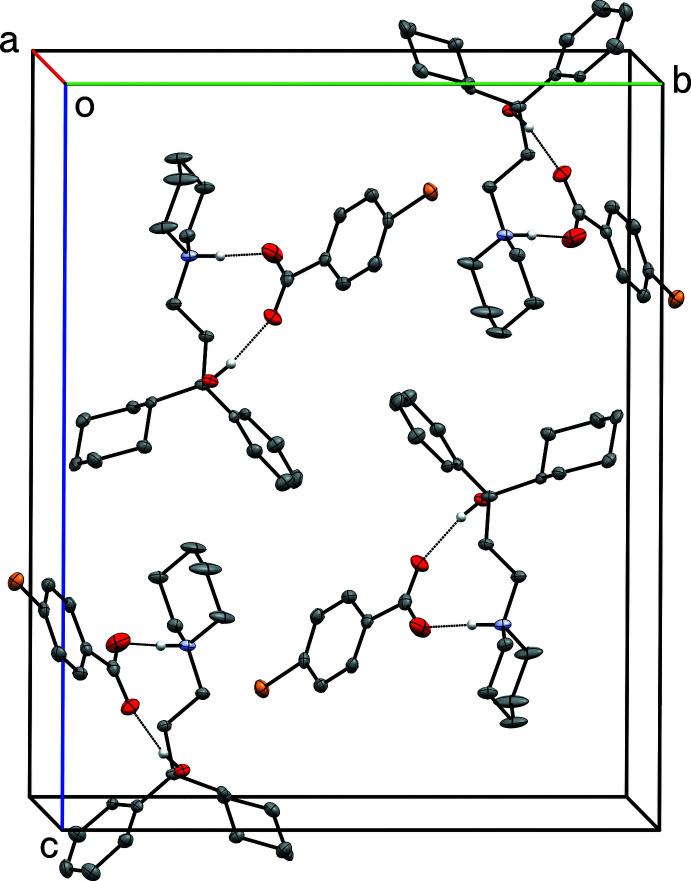
A partial packing plot of **III**, viewed approximately down the *a*-axis. Hydrogen bonds are drawn as dotted lines. Hydrogen atoms not involved in hydrogen bonds are omitted.

**Figure 10:**
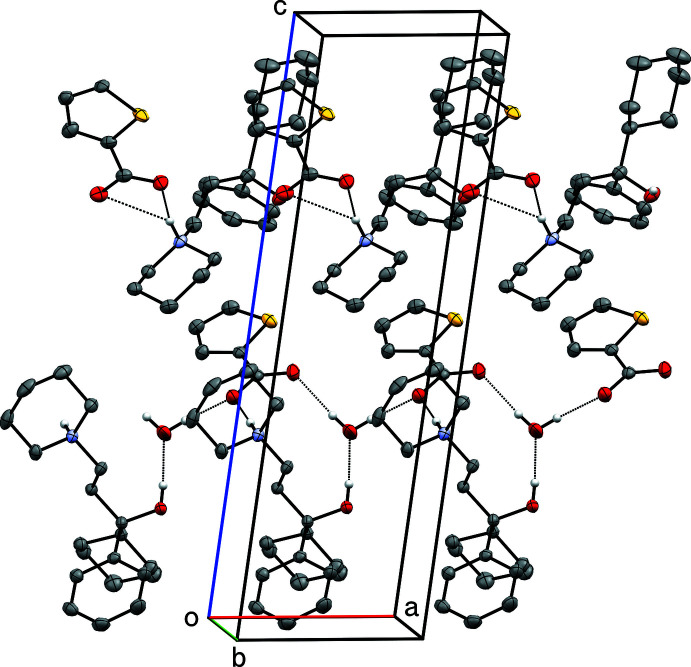
A partial packing plot of **IV**, viewed approximately down the *b*-axis. Hydrogen bonds are drawn as dotted lines. Hydrogen atoms not involved in hydrogen bonds are omitted.

As a result of having two cation–anion pairs and a water mol­ecule in the asymmetric unit, the packing in **IV** is by far the most complex of the four structures. Its hydrogen-bonding patterns are quite different from **I**, **II**, or **III**. In fact the hydrogen-bonding motifs involving the ‘*A*–*C*’ and ‘*B*–*D*’ cation–anion pairs are themselves distinct. For the ‘*A*’ cation, the N1*A*—H1*NA* group is an asymmetric bifurcated hydrogen-bond donor to O1*C* and O2*C* [*d*_D⋯A_ = 2.7084 (17) and 3.3355 (18) Å, respectively]. The hydroxyl group of cation *A* forms a hydrogen bond (as donor) to O2*C* of a translation-related (*via x* + 1, *y*, *z*) anion. These combine to form *A*–*C* cation–anion chains that extend parallel to the *a*-axis (Fig. 10 ▸, upper chain). The *B*–*D* cation–anion pair plus the water mol­ecule form an 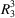 (12) hydrogen-bonded ring motif that includes N1*B*—H1*NB*⋯O1*D*, O1*B*—H1*OB*⋯O1*W*, and O1*W*—H2*W*1⋯O2*D* within the chosen asymmetric unit (Table 5 ▸, Figs. 4 ▸ and 10 ▸). The water mol­ecule also forms a hydrogen bond to O1*D* of a translation-related (*x* + 1, *y*, *z*) anion. The net result of these hydrogen bonds are cation–anion–water chains that also propagate along the *a*-axis direction (Fig. 10 ▸, lower chain). The only contacts between these two types of chain are weak (Table 5 ▸).

## 4. Database survey

A search within the Cambridge Structural Database (CSD, v5.43 including all updates through November 2022; Groom *et al.*, 2016 ▸) for an unsubstituted trihexyphenidyl structure fragment returned 16 hits, but only five of them bear any similarity to the trihexyphenidylium cation in **I**, **II**, **III**, and **IV**. CSD entry THEXPL (Camerman & Camerman, 1972 ▸) is a single-crystal structure of neutral trihexyphenidyl. Refcode KUZDIT (Maccaroni *et al.*, 2010 ▸) is trihexyphenidyl hydro­chloride, obtained *via* powder diffraction, and GODJAN (Shaibah *et al.*, 2019 ▸) is a single-crystal study of the trihexyphenidylium 3,5-di­nitro­benzoate salt. The remaining two structures are PCYDIN10 (Camerman & Camerman, 1971 ▸) and DODWAU (Tacke *et al.*, 1986 ▸). The former is the anti-psychotic medication procyclidine hydro­chloride, which has a pyrrolidinium ring in place of the piperidinium ring in **I**, **II**, **III**, and **IV**. The latter is (*R*)-tricyclamol iodide, which has an *N*-methyl-pyrrolidinium ring.

## 5. Crystallization

A solution of commercially available trihexyphenidyl (a gift from RL Fine Chem., Bengaluru, India) (150 mg, 0.50 mol) in methanol (10 ml) was mixed with equimolar solutions of the appropriate acids in methanol (10 ml) *viz.*, 4-nitro­benzoic acid (84 mg, 0.50 mol) for **I**, and in methanol (5 ml) and aceto­nitrile (5 ml) for 4-hy­droxy­benzoic acid (69 mg, 0.5 mol) (**II**), 4-bromo­benzoic acid (101 mg, 0.50 mol) (**III**) and thio­phene-2-carb­oxy­lic acid (64 mg, 0.50 mol) (**IV**). The resulting solutions were stirred for 30 minutes at 333 K and allowed to stand at room temperature. X-ray quality crystals were formed on slow evaporation over the course of a week for all of the compounds. The melting points were 409–411 K (**I**), 425–427 K (**II**), 395–396 K and (**III**) 368–369 K (**IV**).

## 6. Refinement

Crystal data, data collection, and refinement statistics are given in Table 6 ▸. For all structures, diffraction data were collected with the crystals at 90 K. Non-disordered hydrogen atoms were located in difference-Fourier maps. Those bound to nitro­gen or oxygen were refined freely, but carbon-bound hydrogens were included using riding models with constrained distances of 0.95 Å (C*sp* ^2^—H), 0.99 Å (*R*_2_CH_2_), and 1.00 Å (*R*_3_CH) using *U*_iso_(H) values constrained to 1.2*U*_eq_ of the attached carbon atom. Cation disorder in **I** and **III** was modelled using similar combinations of restraints (*SHELXL* commands SADI, SAME, DFIX, FLAT) and constraints (*SHELXL* command EADP). Disorder of the thio­phene-2-carboxyl­ate anions in **IV** corresponded to a ∼180° flip of the thio­phene ring, which is common for thio­phene, and was modelled using geometry restraints (SAME and FLAT) and displacement parameter constraints (EADP). Structure **II** was twinned by non-merohedry, corresponding to a twofold rotation about the real-space direction [403]. Diffraction data were integrated using two orientation matrices and scaled/merged following standard procedures (see *e.g.* Sevvana *et al.*, 2019 ▸), and the model refined against both twin components in the usual manner (*SHELXL*-format HKLF 5 datafile and a BASF parameter to define their relative volume fractions).

## Supplementary Material

Crystal structure: contains datablock(s) I, II, III, IV, global. DOI: 10.1107/S2056989023005960/vm2286sup1.cif

Structure factors: contains datablock(s) I. DOI: 10.1107/S2056989023005960/vm2286Isup2.hkl

Structure factors: contains datablock(s) II. DOI: 10.1107/S2056989023005960/vm2286IIsup3.hkl

Structure factors: contains datablock(s) III. DOI: 10.1107/S2056989023005960/vm2286IIIsup4.hkl

Structure factors: contains datablock(s) IV. DOI: 10.1107/S2056989023005960/vm2286IVsup5.hkl

Supporting information file. DOI: 10.1107/S2056989023005960/vm2286Isup6.cml

Supporting information file. DOI: 10.1107/S2056989023005960/vm2286IIsup7.cml

Supporting information file. DOI: 10.1107/S2056989023005960/vm2286IIIsup8.cml

Supporting information file. DOI: 10.1107/S2056989023005960/vm2286IVsup9.cml

CCDC references: 2280174, 2280173, 2280172, 2280171

Additional supporting information:  crystallographic information; 3D view; checkCIF report

## Figures and Tables

**Figure 2:**
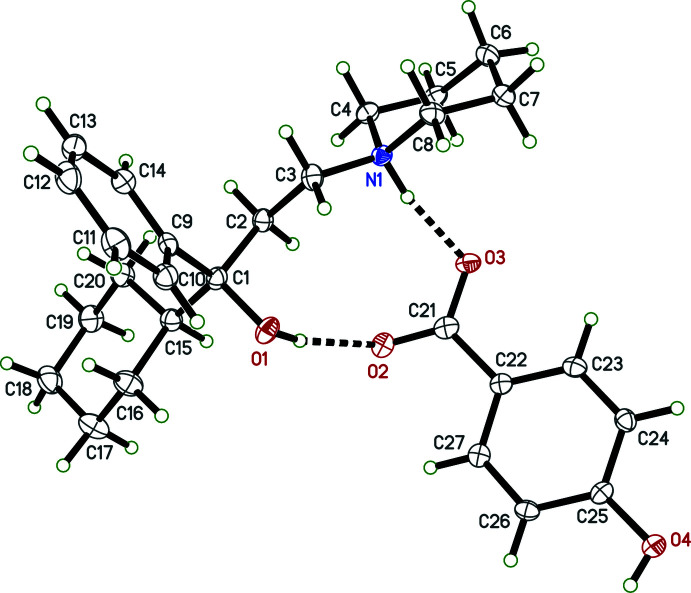
An ellipsoid plot (50%) probability of **II**. Hydrogen bonds are shown as dashed lines.

**Figure 3:**
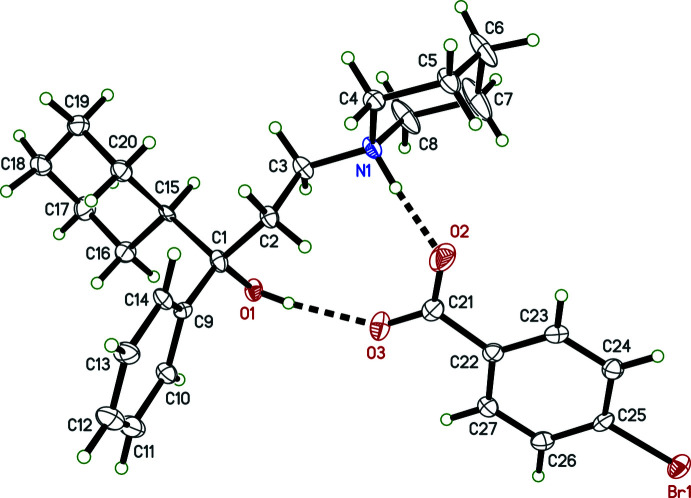
An ellipsoid plot (50%) probability of **III**. Hydrogen bonds are shown as dashed lines. To enhance clarity, only one component of disorder for the cation is shown.

**Table 1:** Conformation-defining torsion angles (°) for trihexyphenyl­idium cations in **I**, **II**, **III**, **IV** The primed (’) atoms in **I** are **III** are for the second disorder component.

*Torsion*	**I**	**II**	**III**	**IVa**	**IVb**
O1—C1—C2—C3	−60.52 (14)	−60.92 (18)	54.5 (2)	−72.04 (17)	−43.38 (16)
C1—C2—C3—N1	152.29 (11)	147.37 (15)	−152.27 (17)	140.13 (14)	−179.42 (12)
C2—C3—N1—C4	59.11 (15)	58.13 (19)	−70.0 (2)	154.13 (13)	83.03 (15)
O1—C1—C9—C10	−25.1 (5)	−13.2 (2)	−23.8 (2)	−20.6 (2)	−9.16 (19)
O1—C1—C15—C16	49.4 (7)	58.47 (19)	57.2 (6)	−72.32 (18)	61.74 (16)
O1—C1—C9′—C10′	17.5 (8)	-	21.4 (4)	-	-
O1—C1—C15′—C16′	168.6 (4)	-	178.8 (5)	-	-

**Table 2:** Hydrogen-bond geometry (Å, °) for **I** [Chem scheme1]

*D*—H⋯*A*	*D*—H	H⋯*A*	*D*⋯*A*	*D*—H⋯*A*
O1—H1*O*⋯O3	0.88 (2)	1.90 (2)	2.7527 (15)	164.5 (19)
N1—H1*N*⋯O2	1.01 (2)	1.65 (2)	2.6618 (15)	172.6 (18)
C4—H4*B*⋯O1^i^	0.99	2.57	3.2972 (17)	130

Symmetry code: (i) 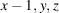 .

**Table 3:** Hydrogen-bond geometry (Å, °) for **II** [Chem scheme1]

*D*—H⋯*A*	*D*—H	H⋯*A*	*D*⋯*A*	*D*—H⋯*A*
O1—H1*O*⋯O2	0.88 (3)	1.94 (3)	2.8068 (18)	165 (2)
N1—H1*N*⋯O3	0.94 (2)	1.76 (2)	2.6908 (19)	169.4 (17)
C2—H2*A*⋯O2	0.99	2.57	3.277 (2)	129
C4—H4*B*⋯O1^i^	0.99	2.40	3.278 (2)	148
C7—H7*A*⋯O3	0.99	2.64	3.314 (2)	126
O4—H4*O*⋯O3^ii^	0.82 (2)	1.84 (3)	2.6633 (18)	176 (3)
C26—H26⋯O3^ii^	0.95	2.62	3.281 (2)	127

Symmetry codes: (i) 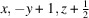 ; (ii) 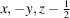 .

**Table 4:** Hydrogen-bond geometry (Å, °) for **III** [Chem scheme1]

*D*—H⋯*A*	*D*—H	H⋯*A*	*D*⋯*A*	*D*—H⋯*A*
N1—H1*N*⋯O2	0.95 (2)	1.67 (3)	2.606 (3)	172 (2)
O1—H1*O*⋯O3	0.81 (3)	1.94 (3)	2.733 (2)	167 (3)
C3—H3*A*⋯Br1^i^	0.99	2.85	3.739 (2)	149
C4—H4*A*⋯O3^ii^	0.99	2.39	3.348 (3)	162

Symmetry codes: (i) 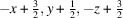 ; (ii) 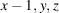 .

**Table 5:** Hydrogen-bond geometry (Å, °) for **IV** [Chem scheme1]

*D*—H⋯*A*	*D*—H	H⋯*A*	*D*⋯*A*	*D*—H⋯*A*
O1*A*—H1*OA*⋯O2*C* ^i^	1.00 (2)	1.72 (2)	2.7143 (16)	172.3 (18)
N1*A*—H1*NA*⋯O1*C*	0.933 (18)	1.793 (18)	2.7084 (17)	166.4 (16)
N1*A*—H1*NA*⋯O2*C*	0.933 (18)	2.606 (18)	3.3355 (18)	135.4 (14)
C4*A*—H4*AB*⋯O1*A* ^ii^	0.99	2.51	3.407 (2)	150
C5*A*—H5*AA*⋯O2*D*	0.99	2.54	3.358 (2)	139
C8*A*—H8*AA*⋯O2*C* ^i^	0.99	2.29	3.283 (2)	177
O1*B*—H1*OB*⋯O1*W*	0.88 (2)	1.85 (2)	2.7186 (18)	169.5 (18)
N1*B*—H1*NB*⋯O1*D*	0.955 (18)	1.700 (18)	2.6497 (17)	172.8 (16)
C3*B*—H3*BB*⋯O1*W*	0.99	2.61	3.295 (2)	126
O1*W*—H1*W*1⋯O1*D* ^i^	0.84 (3)	1.86 (3)	2.6873 (18)	171 (2)
O1*W*—H2*W*1⋯O2*D*	0.81 (3)	1.98 (3)	2.775 (2)	171 (3)

Symmetry codes: (i) 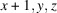 ; (ii) 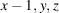 .

**Table 6:** Experimental details

	**I**	**II**	**III**	**IV**
Crystal data				
Chemical formula	C_20_H_32_NO^+^·C_7_H_4_NO_4_^−^	C_20_H_32_NO^+^·C_7_H_5_O^−^	C_20_H_32_NO^+^·C_7_H_4_BrO_2_^−^	2C_20_H_32_NO^+^·2C_5_H_3_O_2_S^−^·H_2_O
*M*_r_	468.58	439.57	502.48	877.21
Crystal system, space group	Triclinic, *P* 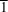	Monoclinic, *C*2/*c*	Monoclinic, *P*2_1_/*n*	Triclinic, *P* 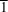
Temperature (K)	90	90	90	90
*a*, *b*, *c* (Å)	6.2568 (5), 11.7542 (14), 16.9162 (19)	45.098 (2), 8.5314 (5), 12.3516 (6)	6.2422 (4), 17.8126 (14), 21.9938 (19)	6.2765 (3), 18.5390 (13), 20.6383 (14)
α, β, γ (°)	85.329 (3), 79.534 (4), 87.785 (3)	90, 101.789 (2), 90	90, 97.345 (3), 90	89.710 (2), 81.600 (2), 88.977 (2)
*V* (Å^3^)	1219.0 (2)	4652.0 (4)	2425.4 (3)	2375.3 (3)
*Z*	2	8	4	2
Radiation type	Mo *K*α	Mo *K*α	Mo *K*α	Mo *K*α
μ (mm^−1^)	0.09	0.08	1.72	0.16
Crystal size (mm)	0.30 × 0.21 × 0.04	0.25 × 0.20 × 0.04	0.20 × 0.08 × 0.07	0.16 × 0.12 × 0.11

Data collection				
Diffractometer	Bruker D8 Venture dual source	Bruker D8 Venture dual source	Bruker D8 Venture dual source	Bruker D8 Venture dual source
Absorption correction	Multi-scan (*SADABS*; Krause *et al.*, 2015 ▸)	Multi-scan (*TWINABS*; Sheldrick, 2012 ▸)	Multi-scan (*SADABS*; Krause *et al.*, 2015 ▸)	Multi-scan (*SADABS*; Krause *et al.*, 2015 ▸)
*T*_min_, *T*_max_	0.907, 0.959	0.706, 0.959	0.740, 0.862	0.908, 0.959
No. of measured, independent and observed [*I* > 2σ(*I*)] reflections	35646, 5590, 4825	5327, 5327, 4543	40844, 5566, 4634	78578, 10922, 8540
*R*_int_	0.041	0.062	0.049	0.049
(sin θ/λ)_max_ (Å^−1^)	0.651	0.650	0.650	0.650

Refinement				
*R*[*F* ^2^ > 2σ(*F* ^2^)], *wR*(*F* ^2^), *S*	0.047, 0.107, 1.11	0.041, 0.090, 1.03	0.037, 0.074, 1.13	0.042, 0.101, 1.03
No. of reflections	5590	5327	5566	10922
No. of parameters	355	303	334	603
No. of restraints	68	0	68	168
H-atom treatment	H atoms treated by a mixture of independent and constrained refinement	H atoms treated by a mixture of independent and constrained refinement	H atoms treated by a mixture of independent and constrained refinement	H atoms treated by a mixture of independent and constrained refinement
Δρ_max_, Δρ_min_ (e Å^−3^)	0.30, −0.19	0.28, −0.28	0.33, −0.38	0.71, −0.30

Computer programs: *APEX3* (Bruker, 2016 ▸), *SHELXT* (Sheldrick, 2015*a* ▸), *SHELXL2019/2* (Sheldrick, 2015*b* ▸), *XP* in *SHELXTL* and *SHELX* (Sheldrick, 2008 ▸), *Mercury* (Macrae *et al.*, 2020 ▸) and *publCIF* (Westrip, 2010 ▸).

## supplementary crystallographic information

### 1-(3-Cyclohexyl-3-hydroxy-3-phenylpropyl)piperidin-1-ium 4-nitrobenzoate (I) Crystal data

**Table:**

C_20_H_32_NO^+^·C_7_H_4_NO_4_^−^	*Z* = 2
*M**_r_* = 468.58	*F*(000) = 504
Triclinic, *P*1	*D*_x_ = 1.277 Mg m^−^^3^
*a* = 6.2568 (5) Å	Mo *K*α radiation, λ = 0.71073 Å
*b* = 11.7542 (14) Å	Cell parameters from 9957 reflections
*c* = 16.9162 (19) Å	θ = 2.2–27.5°
α = 85.329 (3)°	µ = 0.09 mm^−^^1^
β = 79.534 (4)°	*T* = 90 K
γ = 87.785 (3)°	Plate, colourless
*V* = 1219.0 (2) Å^3^	0.30 × 0.21 × 0.04 mm

### 1-(3-Cyclohexyl-3-hydroxy-3-phenylpropyl)piperidin-1-ium 4-nitrobenzoate (I) Data collection

**Table:**

Bruker D8 Venture dual source diffractometer	5590 independent reflections
Radiation source: microsource	4825 reflections with *I* > 2σ(*I*)
Detector resolution: 7.41 pixels mm^-1^	*R*_int_ = 0.041
φ and ω scans	θ_max_ = 27.6°, θ_min_ = 2.1°
Absorption correction: multi-scan (*SADABS*; Krause *et al.*, 2015)	*h* = −8→7
*T*_min_ = 0.907, *T*_max_ = 0.959	*k* = −15→15
35646 measured reflections	*l* = −21→21

### 1-(3-Cyclohexyl-3-hydroxy-3-phenylpropyl)piperidin-1-ium 4-nitrobenzoate (I) Refinement

**Table:**

Refinement on *F*^2^	Secondary atom site location: difference Fourier map
Least-squares matrix: full	Hydrogen site location: mixed
*R*[*F*^2^ > 2σ(*F*^2^)] = 0.047	H atoms treated by a mixture of independent and constrained refinement
*wR*(*F*^2^) = 0.107	*w* = 1/[σ^2^(*F*_o_^2^) + (0.0274*P*)^2^ + 0.7788*P*] where *P* = (*F*_o_^2^ + 2*F*_c_^2^)/3
*S* = 1.11	(Δ/σ)_max_ < 0.001
5590 reflections	Δρ_max_ = 0.30 e Å^−^^3^
355 parameters	Δρ_min_ = −0.19 e Å^−^^3^
68 restraints	Extinction correction: SHELXL-2019/2 (Sheldrick 2008), Fc^*^=kFc[1+0.001xFc^2^λ^3^/sin(2θ)]^-1/4^
Primary atom site location: structure-invariant direct methods	Extinction coefficient: 0.0101 (18)

### 1-(3-Cyclohexyl-3-hydroxy-3-phenylpropyl)piperidin-1-ium 4-nitrobenzoate (I) Special details

**Table:**

Experimental. The crystal was mounted using polyisobutene oil on the tip of a fine glass fibre, which was fastened in a copper mounting pin with electrical solder. It was placed directly into the cold gas stream of a liquid-nitrogen based cryostat (Hope, 1994; Parkin & Hope, 1998). Diffraction data were collected with the crystal at 90K, which is standard practice in this laboratory for the majority of flash-cooled crystals.
Geometry. All esds (except the esd in the dihedral angle between two l.s. planes) are estimated using the full covariance matrix. The cell esds are taken into account individually in the estimation of esds in distances, angles and torsion angles; correlations between esds in cell parameters are only used when they are defined by crystal symmetry. An approximate (isotropic) treatment of cell esds is used for estimating esds involving l.s. planes.
Refinement. Refinement progress was checked using *Platon* (Spek, 2020) and by an *R*-tensor (Parkin, 2000). The final model was further checked with the IUCr utility *checkCIF*.

### 1-(3-Cyclohexyl-3-hydroxy-3-phenylpropyl)piperidin-1-ium 4-nitrobenzoate (I) Fractional atomic coordinates and isotropic or equivalent isotropic displacement parameters (Å^2^)

**Table:**

	*x*	*y*	*z*	*U*_iso_*/*U*_eq_	Occ. (<1)
O1	0.82422 (17)	0.32282 (9)	0.66770 (6)	0.0204 (2)	
H1O	0.781 (3)	0.3907 (18)	0.6831 (12)	0.037 (5)*	
N1	0.28880 (18)	0.3218 (1)	0.83935 (7)	0.0149 (2)	
H1N	0.343 (3)	0.4029 (18)	0.8331 (12)	0.040 (5)*	
C2	0.4351 (2)	0.30509 (12)	0.69168 (8)	0.0173 (3)	
H2A	0.415565	0.389099	0.691340	0.021*	
H2B	0.312841	0.274844	0.670545	0.021*	
C3	0.4311 (2)	0.25491 (12)	0.77785 (8)	0.0185 (3)	
H3A	0.379240	0.175688	0.782916	0.022*	
H3B	0.581117	0.251798	0.789138	0.022*	
C4	0.0570 (2)	0.33002 (12)	0.82710 (9)	0.0191 (3)	
H4A	−0.000299	0.252326	0.828888	0.023*	
H4B	0.049986	0.368697	0.773374	0.023*	
C5	−0.0824 (2)	0.39645 (12)	0.89201 (9)	0.0223 (3)	
H5A	−0.235727	0.397598	0.884423	0.027*	
H5B	−0.033901	0.476326	0.886619	0.027*	
C6	−0.0675 (3)	0.34350 (13)	0.97607 (9)	0.0260 (3)	
H6A	−0.153718	0.390390	1.017119	0.031*	
H6B	−0.127707	0.265915	0.983520	0.031*	
C7	0.1686 (3)	0.33709 (13)	0.98665 (8)	0.0233 (3)	
H7A	0.224705	0.415231	0.983490	0.028*	
H7B	0.179046	0.299809	1.040486	0.028*	
C8	0.3055 (2)	0.26997 (12)	0.92196 (8)	0.0187 (3)	
H8A	0.459291	0.268557	0.928976	0.022*	
H8B	0.256145	0.190218	0.927814	0.022*	
C1	0.6509 (2)	0.27540 (12)	0.63673 (8)	0.0164 (3)	
C9	0.6913 (11)	0.1497 (8)	0.6352 (4)	0.0153 (7)	0.503 (4)
C10	0.9061 (5)	0.1091 (3)	0.6302 (2)	0.0200 (6)	0.503 (4)
H10	1.017442	0.161637	0.630723	0.024*	0.503 (4)
C11	0.9611 (8)	−0.0066 (3)	0.6246 (3)	0.0202 (8)	0.503 (4)
H11	1.107859	−0.032621	0.621441	0.024*	0.503 (4)
C12	0.7960 (9)	−0.0841 (6)	0.6235 (3)	0.0200 (8)	0.503 (4)
H12	0.830948	−0.163027	0.619228	0.024*	0.503 (4)
C13	0.5802 (7)	−0.0449 (4)	0.6288 (3)	0.0217 (8)	0.503 (4)
H13	0.468533	−0.097386	0.628653	0.026*	0.503 (4)
C14	0.5286 (14)	0.0713 (6)	0.6344 (5)	0.0185 (6)	0.503 (4)
H14	0.381829	0.097303	0.637796	0.022*	0.503 (4)
C15	0.639 (2)	0.3364 (10)	0.5511 (7)	0.0156 (12)	0.503 (4)
H15	0.595441	0.417765	0.559790	0.019*	0.503 (4)
C16	0.863 (2)	0.3384 (9)	0.4978 (8)	0.0172 (13)	0.503 (4)
H16A	0.914545	0.259178	0.488027	0.021*	0.503 (4)
H16B	0.967116	0.373263	0.525847	0.021*	0.503 (4)
C17	0.858 (2)	0.4059 (9)	0.4174 (7)	0.0191 (5)	0.503 (4)
H17A	0.818716	0.486717	0.426845	0.023*	0.503 (4)
H17B	1.004414	0.403480	0.383390	0.023*	0.503 (4)
C18	0.695 (2)	0.3579 (7)	0.3737 (6)	0.0202 (12)	0.503 (4)
H18A	0.740979	0.279132	0.360210	0.024*	0.503 (4)
H18B	0.690913	0.404932	0.322736	0.024*	0.503 (4)
C19	0.471 (2)	0.3569 (10)	0.4260 (8)	0.0188 (12)	0.503 (4)
H19A	0.368218	0.321764	0.397780	0.023*	0.503 (4)
H19B	0.420477	0.436353	0.435100	0.023*	0.503 (4)
C20	0.475 (2)	0.2905 (11)	0.5065 (8)	0.0191 (10)	0.503 (4)
H20A	0.511136	0.209320	0.497424	0.023*	0.503 (4)
H20B	0.328551	0.294531	0.540256	0.023*	0.503 (4)
C9'	0.662 (2)	0.3164 (9)	0.5487 (7)	0.0156 (12)	0.497 (4)
C10'	0.851 (2)	0.3635 (8)	0.5021 (8)	0.0172 (13)	0.497 (4)
H10'	0.973969	0.372373	0.526510	0.021*	0.497 (4)
C11'	0.862 (2)	0.3977 (9)	0.4201 (7)	0.0191 (5)	0.497 (4)
H11'	0.991624	0.429451	0.389556	0.023*	0.497 (4)
C12'	0.682 (2)	0.3850 (7)	0.3834 (6)	0.0202 (12)	0.497 (4)
H12'	0.688241	0.407963	0.327914	0.024*	0.497 (4)
C13'	0.492 (2)	0.3382 (10)	0.4292 (8)	0.0188 (12)	0.497 (4)
H13'	0.369144	0.329390	0.404641	0.023*	0.497 (4)
C14'	0.482 (2)	0.3043 (11)	0.5110 (8)	0.0191 (10)	0.497 (4)
H14'	0.352773	0.272635	0.541527	0.023*	0.497 (4)
C15'	0.7100 (12)	0.1425 (8)	0.6432 (4)	0.0153 (7)	0.497 (4)
H15'	0.730895	0.122954	0.699947	0.018*	0.497 (4)
C16'	0.5296 (15)	0.0665 (6)	0.6297 (6)	0.0200 (6)	0.497 (4)
H16C	0.391453	0.087722	0.664630	0.024*	0.497 (4)
H16D	0.509202	0.078531	0.572899	0.024*	0.497 (4)
C17'	0.5852 (7)	−0.0584 (4)	0.6487 (3)	0.0202 (8)	0.497 (4)
H17C	0.467720	−0.106094	0.638287	0.024*	0.497 (4)
H17D	0.594430	−0.071330	0.706574	0.024*	0.497 (4)
C18'	0.7999 (9)	−0.0947 (6)	0.5981 (3)	0.0200 (8)	0.497 (4)
H18C	0.838653	−0.173790	0.616098	0.024*	0.497 (4)
H18D	0.783465	−0.093545	0.540911	0.024*	0.497 (4)
C19'	0.9794 (8)	−0.0169 (4)	0.6051 (4)	0.0217 (8)	0.497 (4)
H19C	1.110868	−0.036668	0.566067	0.026*	0.497 (4)
H19D	1.014763	−0.029729	0.659896	0.026*	0.497 (4)
C20'	0.9217 (5)	0.1084 (2)	0.5897 (3)	0.0185 (6)	0.497 (4)
H20C	0.907689	0.124037	0.532398	0.022*	0.497 (4)
H20D	1.040472	0.155317	0.599858	0.022*	0.497 (4)
O2	0.40809 (17)	0.53766 (9)	0.83371 (6)	0.0228 (2)	
O3	0.71358 (18)	0.52200 (9)	0.74198 (6)	0.0271 (3)	
O4	1.0546 (2)	1.02209 (11)	0.85879 (11)	0.0526 (4)	
O5	0.7218 (2)	1.07163 (11)	0.90394 (11)	0.0524 (4)	
N2	0.8591 (2)	1.00387 (12)	0.87272 (10)	0.0365 (4)	
C21	0.5869 (2)	0.57318 (12)	0.79342 (8)	0.0179 (3)	
C22	0.6545 (2)	0.68961 (11)	0.81174 (8)	0.0170 (3)	
C23	0.8706 (2)	0.72132 (12)	0.78779 (8)	0.0191 (3)	
H23	0.972057	0.671709	0.757832	0.023*	
C24	0.9384 (2)	0.82466 (12)	0.80738 (9)	0.0214 (3)	
H24	1.085757	0.846162	0.791836	0.026*	
C25	0.7863 (3)	0.89562 (12)	0.85005 (10)	0.0246 (3)	
C26	0.5694 (3)	0.86797 (13)	0.87355 (11)	0.0287 (4)	
H26	0.467630	0.918967	0.901992	0.034*	
C27	0.5057 (2)	0.76343 (12)	0.85422 (9)	0.0223 (3)	
H27	0.358403	0.742036	0.870235	0.027*	

### 1-(3-Cyclohexyl-3-hydroxy-3-phenylpropyl)piperidin-1-ium 4-nitrobenzoate (I) Atomic displacement parameters (Å^2^)

**Table:**

	*U*^11^	*U*^22^	*U*^33^	*U*^12^	*U*^13^	*U*^23^
O1	0.0231 (5)	0.0180 (5)	0.0231 (5)	−0.0037 (4)	−0.0100 (4)	−0.0046 (4)
N1	0.0161 (6)	0.0155 (6)	0.0135 (5)	−0.0013 (4)	−0.0030 (4)	−0.0018 (4)
C2	0.0211 (7)	0.0168 (7)	0.0149 (6)	0.0012 (5)	−0.0049 (5)	−0.0026 (5)
C3	0.0202 (7)	0.0192 (7)	0.0157 (7)	0.0044 (5)	−0.0022 (5)	−0.0031 (5)
C4	0.0171 (7)	0.0188 (7)	0.0229 (7)	−0.0018 (5)	−0.0063 (5)	−0.0035 (5)
C5	0.0186 (7)	0.0185 (7)	0.0281 (8)	0.0008 (5)	0.0001 (6)	−0.0006 (6)
C6	0.0306 (8)	0.0181 (7)	0.0240 (8)	0.0012 (6)	0.0080 (6)	0.0008 (6)
C7	0.0370 (9)	0.0178 (7)	0.0137 (7)	−0.0004 (6)	−0.0010 (6)	0.0003 (5)
C8	0.0233 (7)	0.0183 (7)	0.0145 (6)	−0.0008 (5)	−0.0043 (5)	0.0009 (5)
C1	0.0177 (6)	0.0171 (7)	0.0155 (6)	−0.0039 (5)	−0.0045 (5)	−0.0028 (5)
C9	0.0155 (11)	0.0162 (11)	0.0147 (12)	−0.0003 (9)	−0.0040 (9)	−0.0019 (9)
C10	0.0188 (13)	0.0194 (14)	0.0221 (14)	−0.0033 (10)	−0.0039 (9)	−0.0018 (9)
C11	0.0217 (15)	0.0168 (13)	0.024 (2)	−0.0003 (10)	−0.0081 (11)	−0.0012 (9)
C12	0.0237 (9)	0.0163 (13)	0.020 (3)	0.0006 (8)	−0.0031 (17)	−0.0017 (19)
C13	0.0210 (15)	0.0209 (14)	0.025 (2)	−0.0009 (10)	−0.0069 (11)	−0.0045 (10)
C14	0.0134 (12)	0.0168 (13)	0.0243 (15)	0.0006 (9)	0.0004 (9)	−0.0043 (9)
C15	0.017 (2)	0.013 (3)	0.0174 (8)	−0.001 (2)	−0.0035 (11)	−0.0007 (16)
C16	0.0158 (13)	0.018 (4)	0.0177 (12)	−0.002 (2)	−0.0027 (10)	−0.004 (2)
C17	0.0177 (8)	0.0186 (12)	0.0192 (9)	−0.0011 (8)	0.0009 (6)	−0.0005 (9)
C18	0.0247 (15)	0.019 (4)	0.015 (2)	0.001 (3)	−0.0024 (15)	0.001 (2)
C19	0.0205 (19)	0.018 (3)	0.0185 (10)	0.0010 (18)	−0.0055 (11)	−0.0008 (18)
C20	0.0167 (9)	0.024 (2)	0.0173 (13)	−0.0028 (13)	−0.0033 (8)	−0.0019 (15)
C9'	0.017 (2)	0.013 (3)	0.0174 (8)	−0.001 (2)	−0.0035 (11)	−0.0007 (16)
C10'	0.0158 (13)	0.018 (4)	0.0177 (12)	−0.002 (2)	−0.0027 (10)	−0.004 (2)
C11'	0.0177 (8)	0.0186 (12)	0.0192 (9)	−0.0011 (8)	0.0009 (6)	−0.0005 (9)
C12'	0.0247 (15)	0.019 (4)	0.015 (2)	0.001 (3)	−0.0024 (15)	0.001 (2)
C13'	0.0205 (19)	0.018 (3)	0.0185 (10)	0.0010 (18)	−0.0055 (11)	−0.0008 (18)
C14'	0.0167 (9)	0.024 (2)	0.0173 (13)	−0.0028 (13)	−0.0033 (8)	−0.0019 (15)
C15'	0.0155 (11)	0.0162 (11)	0.0147 (12)	−0.0003 (9)	−0.0040 (9)	−0.0019 (9)
C16'	0.0188 (13)	0.0194 (14)	0.0221 (14)	−0.0033 (10)	−0.0039 (9)	−0.0018 (9)
C17'	0.0217 (15)	0.0168 (13)	0.024 (2)	−0.0003 (10)	−0.0081 (11)	−0.0012 (9)
C18'	0.0237 (9)	0.0163 (13)	0.020 (3)	0.0006 (8)	−0.0031 (17)	−0.0017 (19)
C19'	0.0210 (15)	0.0209 (14)	0.025 (2)	−0.0009 (10)	−0.0069 (11)	−0.0045 (10)
C20'	0.0134 (12)	0.0168 (13)	0.0243 (15)	0.0006 (9)	0.0004 (9)	−0.0043 (9)
O2	0.0253 (5)	0.0184 (5)	0.0243 (5)	−0.0074 (4)	−0.0022 (4)	−0.0011 (4)
O3	0.0324 (6)	0.0253 (6)	0.0235 (6)	−0.0079 (5)	0.0002 (5)	−0.0103 (4)
O4	0.0333 (7)	0.0273 (7)	0.1028 (13)	−0.0086 (6)	−0.0199 (8)	−0.0171 (7)
O5	0.0425 (8)	0.0214 (6)	0.0975 (12)	0.0029 (6)	−0.0154 (8)	−0.0250 (7)
N2	0.0337 (8)	0.0158 (7)	0.0639 (11)	−0.0020 (6)	−0.0171 (7)	−0.0070 (7)
C21	0.0240 (7)	0.0163 (7)	0.0146 (6)	−0.0039 (5)	−0.0069 (5)	0.0007 (5)
C22	0.0223 (7)	0.0151 (6)	0.0145 (6)	−0.0039 (5)	−0.0065 (5)	0.0022 (5)
C23	0.0219 (7)	0.0192 (7)	0.0158 (6)	−0.0026 (5)	−0.0024 (5)	−0.0005 (5)
C24	0.0216 (7)	0.0195 (7)	0.0238 (7)	−0.0057 (6)	−0.0071 (6)	0.0034 (6)
C25	0.0277 (8)	0.0118 (7)	0.0368 (9)	−0.0027 (6)	−0.0127 (7)	−0.0006 (6)
C26	0.0240 (8)	0.0161 (7)	0.0473 (10)	0.0039 (6)	−0.0087 (7)	−0.0067 (7)
C27	0.0178 (7)	0.0182 (7)	0.0317 (8)	−0.0009 (5)	−0.0068 (6)	0.0000 (6)

### 1-(3-Cyclohexyl-3-hydroxy-3-phenylpropyl)piperidin-1-ium 4-nitrobenzoate (I) Geometric parameters (Å, º)

**Table:**

O1—C1	1.4358 (16)	C18—H18A	0.9900
O1—H1O	0.88 (2)	C18—H18B	0.9900
N1—C3	1.4953 (17)	C19—C20	1.516 (7)
N1—C8	1.4982 (17)	C19—H19A	0.9900
N1—C4	1.5004 (17)	C19—H19B	0.9900
N1—H1N	1.01 (2)	C20—H20A	0.9900
C2—C3	1.5235 (18)	C20—H20B	0.9900
C2—C1	1.5396 (19)	C9'—C10'	1.404 (7)
C2—H2A	0.9900	C9'—C14'	1.405 (7)
C2—H2B	0.9900	C10'—C11'	1.401 (6)
C3—H3A	0.9900	C10'—H10'	0.9500
C3—H3B	0.9900	C11'—C12'	1.402 (7)
C4—C5	1.522 (2)	C11'—H11'	0.9500
C4—H4A	0.9900	C12'—C13'	1.400 (7)
C4—H4B	0.9900	C12'—H12'	0.9500
C5—C6	1.522 (2)	C13'—C14'	1.401 (7)
C5—H5A	0.9900	C13'—H13'	0.9500
C5—H5B	0.9900	C14'—H14'	0.9500
C6—C7	1.518 (2)	C15'—C20'	1.522 (7)
C6—H6A	0.9900	C15'—C16'	1.526 (7)
C6—H6B	0.9900	C15'—H15'	1.0000
C7—C8	1.517 (2)	C16'—C17'	1.518 (7)
C7—H7A	0.9900	C16'—H16C	0.9900
C7—H7B	0.9900	C16'—H16D	0.9900
C8—H8A	0.9900	C17'—C18'	1.522 (6)
C8—H8B	0.9900	C17'—H17C	0.9900
C1—C9	1.490 (9)	C17'—H17D	0.9900
C1—C9'	1.517 (11)	C18'—C19'	1.502 (6)
C1—C15	1.576 (11)	C18'—H18C	0.9900
C1—C15'	1.592 (10)	C18'—H18D	0.9900
C9—C10	1.399 (7)	C19'—C20'	1.516 (5)
C9—C14	1.402 (7)	C19'—H19C	0.9900
C10—C11	1.397 (5)	C19'—H19D	0.9900
C10—H10	0.9500	C20'—H20C	0.9900
C11—C12	1.407 (6)	C20'—H20D	0.9900
C11—H11	0.9500	O2—C21	1.2661 (17)
C12—C13	1.399 (6)	O3—C21	1.2431 (18)
C12—H12	0.9500	O4—N2	1.227 (2)
C13—C14	1.398 (6)	O5—N2	1.226 (2)
C13—H13	0.9500	N2—C25	1.4698 (19)
C14—H14	0.9500	C21—C22	1.5197 (19)
C15—C20	1.519 (7)	C22—C27	1.390 (2)
C15—C16	1.521 (7)	C22—C23	1.395 (2)
C15—H15	1.0000	C23—C24	1.386 (2)
C16—C17	1.523 (7)	C23—H23	0.9500
C16—H16A	0.9900	C24—C25	1.382 (2)
C16—H16B	0.9900	C24—H24	0.9500
C17—C18	1.512 (7)	C25—C26	1.387 (2)
C17—H17A	0.9900	C26—C27	1.388 (2)
C17—H17B	0.9900	C26—H26	0.9500
C18—C19	1.515 (7)	C27—H27	0.9500
			
C1—O1—H1O	107.8 (13)	C17—C18—C19	110.3 (6)
C3—N1—C8	109.22 (10)	C17—C18—H18A	109.6
C3—N1—C4	112.50 (11)	C19—C18—H18A	109.6
C8—N1—C4	111.30 (11)	C17—C18—H18B	109.6
C3—N1—H1N	108.1 (11)	C19—C18—H18B	109.6
C8—N1—H1N	109.2 (11)	H18A—C18—H18B	108.1
C4—N1—H1N	106.4 (11)	C18—C19—C20	111.0 (6)
C3—C2—C1	111.20 (11)	C18—C19—H19A	109.4
C3—C2—H2A	109.4	C20—C19—H19A	109.4
C1—C2—H2A	109.4	C18—C19—H19B	109.4
C3—C2—H2B	109.4	C20—C19—H19B	109.4
C1—C2—H2B	109.4	H19A—C19—H19B	108.0
H2A—C2—H2B	108.0	C19—C20—C15	112.1 (7)
N1—C3—C2	113.20 (11)	C19—C20—H20A	109.2
N1—C3—H3A	108.9	C15—C20—H20A	109.2
C2—C3—H3A	108.9	C19—C20—H20B	109.2
N1—C3—H3B	108.9	C15—C20—H20B	109.2
C2—C3—H3B	108.9	H20A—C20—H20B	107.9
H3A—C3—H3B	107.8	C10'—C9'—C14'	118.1 (7)
N1—C4—C5	110.64 (11)	C10'—C9'—C1	121.7 (10)
N1—C4—H4A	109.5	C14'—C9'—C1	120.1 (10)
C5—C4—H4A	109.5	C11'—C10'—C9'	121.3 (7)
N1—C4—H4B	109.5	C11'—C10'—H10'	119.3
C5—C4—H4B	109.5	C9'—C10'—H10'	119.3
H4A—C4—H4B	108.1	C10'—C11'—C12'	119.8 (7)
C4—C5—C6	111.42 (12)	C10'—C11'—H11'	120.1
C4—C5—H5A	109.3	C12'—C11'—H11'	120.1
C6—C5—H5A	109.3	C13'—C12'—C11'	119.5 (7)
C4—C5—H5B	109.3	C13'—C12'—H12'	120.2
C6—C5—H5B	109.3	C11'—C12'—H12'	120.2
H5A—C5—H5B	108.0	C12'—C13'—C14'	120.2 (7)
C7—C6—C5	109.47 (12)	C12'—C13'—H13'	119.9
C7—C6—H6A	109.8	C14'—C13'—H13'	119.9
C5—C6—H6A	109.8	C13'—C14'—C9'	121.0 (7)
C7—C6—H6B	109.8	C13'—C14'—H14'	119.5
C5—C6—H6B	109.8	C9'—C14'—H14'	119.5
H6A—C6—H6B	108.2	C20'—C15'—C16'	109.0 (6)
C8—C7—C6	110.62 (12)	C20'—C15'—C1	114.6 (5)
C8—C7—H7A	109.5	C16'—C15'—C1	114.1 (5)
C6—C7—H7A	109.5	C20'—C15'—H15'	106.1
C8—C7—H7B	109.5	C16'—C15'—H15'	106.1
C6—C7—H7B	109.5	C1—C15'—H15'	106.1
H7A—C7—H7B	108.1	C17'—C16'—C15'	110.7 (6)
N1—C8—C7	111.18 (11)	C17'—C16'—H16C	109.5
N1—C8—H8A	109.4	C15'—C16'—H16C	109.5
C7—C8—H8A	109.4	C17'—C16'—H16D	109.5
N1—C8—H8B	109.4	C15'—C16'—H16D	109.5
C7—C8—H8B	109.4	H16C—C16'—H16D	108.1
H8A—C8—H8B	108.0	C16'—C17'—C18'	111.6 (5)
O1—C1—C9	108.1 (3)	C16'—C17'—H17C	109.3
O1—C1—C9'	110.1 (6)	C18'—C17'—H17C	109.3
O1—C1—C2	108.07 (11)	C16'—C17'—H17D	109.3
C9—C1—C2	112.1 (3)	C18'—C17'—H17D	109.3
C9'—C1—C2	114.3 (5)	H17C—C17'—H17D	108.0
O1—C1—C15	109.0 (5)	C19'—C18'—C17'	111.4 (4)
C9—C1—C15	113.1 (5)	C19'—C18'—H18C	109.4
C2—C1—C15	106.3 (5)	C17'—C18'—H18C	109.4
O1—C1—C15'	102.0 (3)	C19'—C18'—H18D	109.4
C9'—C1—C15'	109.1 (5)	C17'—C18'—H18D	109.4
C2—C1—C15'	112.5 (3)	H18C—C18'—H18D	108.0
C10—C9—C14	118.5 (6)	C18'—C19'—C20'	113.1 (4)
C10—C9—C1	117.6 (5)	C18'—C19'—H19C	109.0
C14—C9—C1	123.8 (5)	C20'—C19'—H19C	109.0
C11—C10—C9	121.7 (5)	C18'—C19'—H19D	109.0
C11—C10—H10	119.1	C20'—C19'—H19D	109.0
C9—C10—H10	119.1	H19C—C19'—H19D	107.8
C10—C11—C12	119.0 (4)	C19'—C20'—C15'	111.7 (4)
C10—C11—H11	120.5	C19'—C20'—H20C	109.3
C12—C11—H11	120.5	C15'—C20'—H20C	109.3
C13—C12—C11	119.9 (5)	C19'—C20'—H20D	109.3
C13—C12—H12	120.0	C15'—C20'—H20D	109.3
C11—C12—H12	120.0	H20C—C20'—H20D	107.9
C14—C13—C12	120.1 (5)	O5—N2—O4	123.33 (14)
C14—C13—H13	119.9	O5—N2—C25	118.52 (14)
C12—C13—H13	119.9	O4—N2—C25	118.15 (14)
C13—C14—C9	120.7 (6)	O3—C21—O2	126.87 (13)
C13—C14—H14	119.7	O3—C21—C22	117.08 (12)
C9—C14—H14	119.7	O2—C21—C22	116.04 (12)
C20—C15—C16	109.9 (6)	C27—C22—C23	119.55 (13)
C20—C15—C1	116.1 (9)	C27—C22—C21	120.94 (13)
C16—C15—C1	110.7 (8)	C23—C22—C21	119.48 (13)
C20—C15—H15	106.5	C24—C23—C22	120.50 (14)
C16—C15—H15	106.5	C24—C23—H23	119.8
C1—C15—H15	106.5	C22—C23—H23	119.7
C15—C16—C17	111.3 (6)	C25—C24—C23	118.38 (14)
C15—C16—H16A	109.4	C25—C24—H24	120.8
C17—C16—H16A	109.4	C23—C24—H24	120.8
C15—C16—H16B	109.4	C24—C25—C26	122.75 (14)
C17—C16—H16B	109.4	C24—C25—N2	118.45 (14)
H16A—C16—H16B	108.0	C26—C25—N2	118.80 (14)
C18—C17—C16	111.1 (6)	C25—C26—C27	117.89 (15)
C18—C17—H17A	109.4	C25—C26—H26	121.1
C16—C17—H17A	109.4	C27—C26—H26	121.1
C18—C17—H17B	109.4	C26—C27—C22	120.92 (14)
C16—C17—H17B	109.4	C26—C27—H27	119.5
H17A—C17—H17B	108.0	C22—C27—H27	119.5
			
C8—N1—C3—C2	−176.78 (11)	C2—C1—C9'—C10'	139.3 (6)
C4—N1—C3—C2	59.11 (15)	C15'—C1—C9'—C10'	−93.7 (7)
C1—C2—C3—N1	152.29 (11)	O1—C1—C9'—C14'	−164.6 (5)
C3—N1—C4—C5	178.76 (11)	C2—C1—C9'—C14'	−42.8 (7)
C8—N1—C4—C5	55.81 (14)	C15'—C1—C9'—C14'	84.1 (7)
N1—C4—C5—C6	−56.25 (16)	C14'—C9'—C10'—C11'	0.0 (3)
C4—C5—C6—C7	56.67 (16)	C1—C9'—C10'—C11'	177.9 (8)
C5—C6—C7—C8	−56.90 (16)	C9'—C10'—C11'—C12'	0.0 (3)
C3—N1—C8—C7	178.38 (12)	C10'—C11'—C12'—C13'	0.0 (6)
C4—N1—C8—C7	−56.81 (15)	C11'—C12'—C13'—C14'	0.0 (8)
C6—C7—C8—N1	57.53 (15)	C12'—C13'—C14'—C9'	0.0 (8)
C3—C2—C1—O1	−60.52 (14)	C10'—C9'—C14'—C13'	0.0 (6)
C3—C2—C1—C9	58.5 (3)	C1—C9'—C14'—C13'	−177.9 (9)
C3—C2—C1—C9'	176.5 (5)	O1—C1—C15'—C20'	−64.7 (6)
C3—C2—C1—C15	−177.4 (5)	C9'—C1—C15'—C20'	51.8 (8)
C3—C2—C1—C15'	51.3 (3)	C2—C1—C15'—C20'	179.7 (4)
O1—C1—C9—C10	−25.0 (5)	O1—C1—C15'—C16'	168.6 (4)
C2—C1—C9—C10	−144.1 (3)	C9'—C1—C15'—C16'	−74.9 (7)
C15—C1—C9—C10	95.7 (7)	C2—C1—C15'—C16'	53.1 (5)
O1—C1—C9—C14	158.2 (4)	C20'—C15'—C16'—C17'	58.7 (7)
C2—C1—C9—C14	39.1 (5)	C1—C15'—C16'—C17'	−171.8 (5)
C15—C1—C9—C14	−81.1 (7)	C15'—C16'—C17'—C18'	−57.6 (7)
C14—C9—C10—C11	0.1 (3)	C16'—C17'—C18'—C19'	53.0 (6)
C1—C9—C10—C11	−176.9 (5)	C17'—C18'—C19'—C20'	−51.2 (6)
C9—C10—C11—C12	0.2 (2)	C18'—C19'—C20'—C15'	54.0 (6)
C10—C11—C12—C13	−0.5 (5)	C16'—C15'—C20'—C19'	−56.6 (6)
C11—C12—C13—C14	0.6 (6)	C1—C15'—C20'—C19'	174.1 (5)
C12—C13—C14—C9	−0.3 (7)	O3—C21—C22—C27	165.70 (13)
C10—C9—C14—C13	0.0 (6)	O2—C21—C22—C27	−15.63 (19)
C1—C9—C14—C13	176.7 (6)	O3—C21—C22—C23	−16.34 (19)
O1—C1—C15—C20	175.6 (5)	O2—C21—C22—C23	162.33 (13)
C9—C1—C15—C20	55.4 (8)	C27—C22—C23—C24	1.2 (2)
C2—C1—C15—C20	−68.1 (6)	C21—C22—C23—C24	−176.75 (13)
O1—C1—C15—C16	49.4 (7)	C22—C23—C24—C25	−0.9 (2)
C9—C1—C15—C16	−70.9 (7)	C23—C24—C25—C26	−0.4 (2)
C2—C1—C15—C16	165.6 (5)	C23—C24—C25—N2	178.42 (14)
C20—C15—C16—C17	55.2 (7)	O5—N2—C25—C24	173.37 (16)
C1—C15—C16—C17	−175.1 (8)	O4—N2—C25—C24	−6.1 (2)
C15—C16—C17—C18	−57.1 (6)	O5—N2—C25—C26	−7.8 (2)
C16—C17—C18—C19	57.1 (7)	O4—N2—C25—C26	172.74 (17)
C17—C18—C19—C20	−56.3 (8)	C24—C25—C26—C27	1.2 (2)
C18—C19—C20—C15	56.1 (8)	N2—C25—C26—C27	−177.59 (15)
C16—C15—C20—C19	−55.1 (7)	C25—C26—C27—C22	−0.8 (2)
C1—C15—C20—C19	178.3 (8)	C23—C22—C27—C26	−0.4 (2)
O1—C1—C9'—C10'	17.5 (8)	C21—C22—C27—C26	177.57 (14)

### 1-(3-Cyclohexyl-3-hydroxy-3-phenylpropyl)piperidin-1-ium 4-nitrobenzoate (I) Hydrogen-bond geometry (Å, º)

**Table:**

*D*—H···*A*	*D*—H	H···*A*	*D*···*A*	*D*—H···*A*
O1—H1*O*···O3	0.88 (2)	1.90 (2)	2.7527 (15)	164.5 (19)
N1—H1*N*···O2	1.01 (2)	1.65 (2)	2.6618 (15)	172.6 (18)
C4—H4*B*···O1^i^	0.99	2.57	3.2972 (17)	130

Symmetry code: (i) *x*−1, *y*, *z*.

### 1-(3-Cyclohexyl-3-hydroxy-3-phenylpropyl)piperidin-1-ium 4-hydroxybenzoate (II) Crystal data

**Table:**

C_20_H_32_NO^+^·C_7_H_5_O^−^	*F*(000) = 1904
*M**_r_* = 439.57	*D*_x_ = 1.255 Mg m^−^^3^
Monoclinic, *C*2/*c*	Mo *K*α radiation, λ = 0.71073 Å
*a* = 45.098 (2) Å	Cell parameters from 9845 reflections
*b* = 8.5314 (5) Å	θ = 2.4–27.5°
*c* = 12.3516 (6) Å	µ = 0.08 mm^−^^1^
β = 101.789 (2)°	*T* = 90 K
*V* = 4652.0 (4) Å^3^	Plate, colourless
*Z* = 8	0.25 × 0.20 × 0.04 mm

### 1-(3-Cyclohexyl-3-hydroxy-3-phenylpropyl)piperidin-1-ium 4-hydroxybenzoate (II) Data collection

**Table:**

Bruker D8 Venture dual source diffractometer	5327 independent reflections
Radiation source: microsource	4543 reflections with *I* > 2σ(*I*)
Detector resolution: 7.41 pixels mm^-1^	*R*_int_ = 0.062
φ and ω scans	θ_max_ = 27.5°, θ_min_ = 2.4°
Absorption correction: multi-scan (*TWINABS*; Sheldrick, 2012)	*h* = −58→57
*T*_min_ = 0.706, *T*_max_ = 0.959	*k* = 0→11
5327 measured reflections	*l* = 0→16

### 1-(3-Cyclohexyl-3-hydroxy-3-phenylpropyl)piperidin-1-ium 4-hydroxybenzoate (II) Refinement

**Table:**

Refinement on *F*^2^	Secondary atom site location: difference Fourier map
Least-squares matrix: full	Hydrogen site location: mixed
*R*[*F*^2^ > 2σ(*F*^2^)] = 0.041	H atoms treated by a mixture of independent and constrained refinement
*wR*(*F*^2^) = 0.090	*w* = 1/[σ^2^(*F*_o_^2^) + (0.0332*P*)^2^ + 1.3379*P*] where *P* = (*F*_o_^2^ + 2*F*_c_^2^)/3
*S* = 1.03	(Δ/σ)_max_ = 0.001
5327 reflections	Δρ_max_ = 0.28 e Å^−^^3^
303 parameters	Δρ_min_ = −0.28 e Å^−^^3^
0 restraints	Extinction correction: SHELXL-2019/2 (Sheldrick 2008), Fc^*^=kFc[1+0.001xFc^2^λ^3^/sin(2θ)]^-1/4^
Primary atom site location: structure-invariant direct methods	Extinction coefficient: 0.0030 (5)

### 1-(3-Cyclohexyl-3-hydroxy-3-phenylpropyl)piperidin-1-ium 4-hydroxybenzoate (II) Special details

**Table:**

Experimental. The crystal was mounted using polyisobutene oil on the tip of a fine glass fibre, which was fastened in a copper mounting pin with electrical solder. It was placed directly into the cold gas stream of a liquid-nitrogen based cryostat (Hope, 1994; Parkin & Hope, 1998). Diffraction data were collected with the crystal at 90K, which is standard practice in this laboratory for the majority of flash-cooled crystals.
Geometry. All esds (except the esd in the dihedral angle between two l.s. planes) are estimated using the full covariance matrix. The cell esds are taken into account individually in the estimation of esds in distances, angles and torsion angles; correlations between esds in cell parameters are only used when they are defined by crystal symmetry. An approximate (isotropic) treatment of cell esds is used for estimating esds involving l.s. planes.
Refinement. Refined as a two-component twin.

### 1-(3-Cyclohexyl-3-hydroxy-3-phenylpropyl)piperidin-1-ium 4-hydroxybenzoate (II) Fractional atomic coordinates and isotropic or equivalent isotropic displacement parameters (Å^2^)

**Table:**

	*x*	*y*	*z*	*U*_iso_*/*U*_eq_	
O1	0.64503 (3)	0.47657 (16)	0.23197 (10)	0.0197 (3)	
H1O	0.6394 (5)	0.395 (3)	0.267 (2)	0.037 (6)*	
N1	0.58510 (3)	0.60731 (17)	0.40624 (11)	0.0145 (3)	
H1N	0.5832 (4)	0.499 (3)	0.3955 (17)	0.023 (5)*	
C1	0.66057 (4)	0.5819 (2)	0.31582 (14)	0.0168 (3)	
C2	0.64073 (3)	0.5992 (2)	0.40316 (14)	0.0166 (3)	
H2A	0.638047	0.495231	0.435376	0.020*	
H2B	0.651117	0.668315	0.463538	0.020*	
C3	0.60976 (3)	0.6679 (2)	0.35289 (14)	0.0185 (4)	
H3A	0.610726	0.783437	0.360334	0.022*	
H3B	0.604764	0.642710	0.272994	0.022*	
C4	0.59104 (4)	0.6392 (2)	0.52798 (13)	0.0170 (4)	
H4A	0.592245	0.753753	0.541102	0.020*	
H4B	0.610672	0.592309	0.563876	0.020*	
C5	0.56583 (4)	0.5702 (2)	0.57793 (14)	0.0185 (4)	
H5A	0.569629	0.595026	0.657884	0.022*	
H5B	0.565790	0.454786	0.569906	0.022*	
C6	0.53495 (4)	0.6349 (2)	0.52184 (14)	0.0215 (4)	
H6A	0.518871	0.581952	0.552046	0.026*	
H6B	0.534062	0.748444	0.537538	0.026*	
C7	0.52948 (4)	0.6091 (2)	0.39728 (15)	0.0194 (4)	
H7A	0.527524	0.495443	0.381295	0.023*	
H7B	0.510274	0.660366	0.361565	0.023*	
C8	0.55528 (3)	0.6755 (2)	0.34958 (14)	0.0183 (4)	
H8A	0.551751	0.651862	0.269500	0.022*	
H8B	0.555856	0.790859	0.358541	0.022*	
C9	0.66395 (3)	0.7379 (2)	0.25866 (14)	0.0161 (4)	
C10	0.65862 (4)	0.7484 (2)	0.14377 (15)	0.0203 (4)	
H10	0.652558	0.657720	0.100257	0.024*	
C11	0.66205 (4)	0.8899 (2)	0.09203 (16)	0.0240 (4)	
H11	0.658087	0.895399	0.013573	0.029*	
C12	0.67121 (4)	1.0227 (2)	0.15405 (16)	0.0251 (4)	
H12	0.673865	1.118945	0.118511	0.030*	
C13	0.67646 (4)	1.0144 (2)	0.26827 (16)	0.0222 (4)	
H13	0.682632	1.105283	0.311469	0.027*	
C14	0.67272 (4)	0.8732 (2)	0.31970 (15)	0.0192 (4)	
H14	0.676227	0.868889	0.398139	0.023*	
C15	0.69159 (4)	0.5071 (2)	0.36817 (14)	0.0176 (4)	
H15	0.686969	0.402152	0.396860	0.021*	
C16	0.71081 (4)	0.4760 (2)	0.28197 (16)	0.0244 (4)	
H16A	0.716442	0.577069	0.252371	0.029*	
H16B	0.698755	0.415569	0.219800	0.029*	
C17	0.73957 (4)	0.3845 (3)	0.33156 (16)	0.0281 (4)	
H17A	0.733941	0.278419	0.352781	0.034*	
H17B	0.752121	0.372668	0.274999	0.034*	
C18	0.75806 (4)	0.4665 (3)	0.43271 (17)	0.0276 (4)	
H18A	0.775308	0.398936	0.466766	0.033*	
H18B	0.766361	0.565645	0.409679	0.033*	
C19	0.73880 (4)	0.5015 (2)	0.51730 (16)	0.0254 (4)	
H19A	0.750942	0.561859	0.579318	0.030*	
H19B	0.732701	0.401767	0.547398	0.030*	
C20	0.71056 (4)	0.5949 (2)	0.46637 (15)	0.0210 (4)	
H20A	0.716603	0.698106	0.441359	0.025*	
H20B	0.698238	0.613178	0.522950	0.025*	
O2	0.61716 (3)	0.24225 (15)	0.33577 (10)	0.0223 (3)	
O3	0.57253 (2)	0.30007 (14)	0.37676 (10)	0.0182 (3)	
O4	0.53273 (3)	−0.20004 (15)	−0.00636 (10)	0.0197 (3)	
H4O	0.5455 (5)	−0.227 (3)	−0.042 (2)	0.042 (7)*	
C21	0.58953 (4)	0.2187 (2)	0.32616 (14)	0.0166 (3)	
C22	0.57456 (3)	0.0946 (2)	0.24782 (13)	0.0145 (3)	
C23	0.54314 (4)	0.0716 (2)	0.22493 (14)	0.0164 (3)	
H23	0.531016	0.126006	0.267150	0.020*	
C24	0.52947 (4)	−0.0294 (2)	0.14147 (15)	0.0170 (4)	
H24	0.508153	−0.044621	0.127228	0.020*	
C25	0.54707 (4)	−0.10839 (19)	0.07870 (14)	0.0150 (3)	
C26	0.57859 (4)	−0.0913 (2)	0.10326 (14)	0.0165 (3)	
H26	0.590788	−0.148204	0.062676	0.020*	
C27	0.59185 (3)	0.0093 (2)	0.18711 (14)	0.0158 (3)	
H27	0.613258	0.020403	0.203686	0.019*	

### 1-(3-Cyclohexyl-3-hydroxy-3-phenylpropyl)piperidin-1-ium 4-hydroxybenzoate (II) Atomic displacement parameters (Å^2^)

**Table:**

	*U*^11^	*U*^22^	*U*^33^	*U*^12^	*U*^13^	*U*^23^
O1	0.0239 (6)	0.0189 (7)	0.0149 (6)	−0.0060 (5)	0.0005 (5)	−0.0013 (5)
N1	0.0151 (6)	0.0127 (8)	0.0153 (7)	0.0001 (5)	0.0023 (5)	0.0009 (6)
C1	0.0168 (7)	0.0178 (9)	0.0152 (8)	−0.0029 (7)	0.0021 (6)	−0.0023 (7)
C2	0.0160 (8)	0.0182 (9)	0.0152 (8)	−0.0016 (7)	0.0022 (6)	0.0005 (7)
C3	0.0177 (8)	0.0195 (10)	0.0182 (8)	−0.0018 (7)	0.0037 (6)	0.0046 (7)
C4	0.0200 (8)	0.0175 (9)	0.0129 (8)	−0.0006 (7)	0.0018 (6)	−0.0014 (7)
C5	0.0247 (8)	0.0157 (9)	0.0156 (8)	−0.0018 (7)	0.0051 (7)	−0.0011 (7)
C6	0.0193 (8)	0.0247 (10)	0.0220 (9)	0.0003 (7)	0.0082 (7)	−0.0045 (8)
C7	0.0161 (7)	0.0187 (10)	0.0230 (9)	0.0023 (7)	0.0028 (7)	−0.0011 (7)
C8	0.0175 (8)	0.0183 (9)	0.0180 (8)	0.0045 (7)	0.0009 (7)	0.0024 (7)
C9	0.0110 (7)	0.0187 (9)	0.0189 (8)	0.0002 (6)	0.0037 (6)	0.0007 (7)
C10	0.0197 (8)	0.0219 (10)	0.0197 (9)	−0.0018 (7)	0.0054 (7)	−0.0009 (8)
C11	0.0242 (9)	0.0310 (11)	0.0180 (9)	0.0001 (8)	0.0074 (7)	0.0053 (8)
C12	0.0218 (8)	0.0236 (11)	0.0314 (11)	0.0000 (7)	0.0090 (8)	0.0094 (8)
C13	0.0190 (8)	0.0189 (10)	0.0285 (10)	−0.0016 (7)	0.0048 (7)	0.0000 (8)
C14	0.0183 (8)	0.0208 (10)	0.0188 (9)	0.0002 (7)	0.0042 (7)	0.0002 (7)
C15	0.0181 (7)	0.0179 (9)	0.0172 (9)	−0.0001 (7)	0.0043 (7)	0.0005 (7)
C16	0.0245 (9)	0.0300 (11)	0.0190 (9)	0.0056 (8)	0.0053 (7)	0.0007 (8)
C17	0.0251 (9)	0.0336 (12)	0.0269 (10)	0.0092 (8)	0.0085 (8)	0.0006 (9)
C18	0.0185 (8)	0.0301 (12)	0.0340 (11)	0.0035 (7)	0.0049 (8)	0.0031 (9)
C19	0.0199 (8)	0.0295 (11)	0.0241 (10)	0.0007 (8)	−0.0018 (7)	−0.0005 (8)
C20	0.0181 (8)	0.0242 (10)	0.0193 (9)	0.0012 (7)	0.0009 (7)	−0.0019 (7)
O2	0.0210 (6)	0.0204 (7)	0.0252 (7)	−0.0035 (5)	0.0042 (5)	−0.0029 (6)
O3	0.0241 (6)	0.0147 (6)	0.0172 (6)	0.0005 (5)	0.0074 (5)	−0.0018 (5)
O4	0.0187 (6)	0.0197 (7)	0.0202 (6)	−0.0009 (5)	0.0028 (5)	−0.0051 (5)
C21	0.0243 (8)	0.0114 (9)	0.0146 (8)	0.0013 (7)	0.0047 (7)	0.0063 (7)
C22	0.0184 (8)	0.0118 (8)	0.0136 (8)	0.0006 (6)	0.0038 (6)	0.0034 (6)
C23	0.0202 (8)	0.0130 (9)	0.0175 (8)	0.0022 (7)	0.0071 (7)	0.0024 (7)
C24	0.0154 (7)	0.0159 (9)	0.0201 (9)	0.0002 (6)	0.0046 (7)	0.0021 (7)
C25	0.0194 (8)	0.0104 (8)	0.0141 (8)	−0.0003 (6)	0.0010 (6)	0.0025 (6)
C26	0.0191 (8)	0.0148 (9)	0.0163 (8)	0.0037 (6)	0.0052 (7)	0.0015 (7)
C27	0.0170 (7)	0.0146 (9)	0.0151 (8)	0.0008 (7)	0.0014 (7)	0.0040 (7)

### 1-(3-Cyclohexyl-3-hydroxy-3-phenylpropyl)piperidin-1-ium 4-hydroxybenzoate (II) Geometric parameters (Å, º)

**Table:**

O1—C1	1.440 (2)	C13—C14	1.388 (3)
O1—H1O	0.88 (3)	C13—H13	0.9500
N1—C3	1.496 (2)	C14—H14	0.9500
N1—C4	1.497 (2)	C15—C16	1.527 (2)
N1—C8	1.501 (2)	C15—C20	1.530 (2)
N1—H1N	0.94 (2)	C15—H15	1.0000
C1—C9	1.529 (2)	C16—C17	1.530 (2)
C1—C2	1.543 (2)	C16—H16A	0.9900
C1—C15	1.553 (2)	C16—H16B	0.9900
C2—C3	1.525 (2)	C17—C18	1.523 (3)
C2—H2A	0.9900	C17—H17A	0.9900
C2—H2B	0.9900	C17—H17B	0.9900
C3—H3A	0.9900	C18—C19	1.519 (3)
C3—H3B	0.9900	C18—H18A	0.9900
C4—C5	1.519 (2)	C18—H18B	0.9900
C4—H4A	0.9900	C19—C20	1.525 (2)
C4—H4B	0.9900	C19—H19A	0.9900
C5—C6	1.526 (2)	C19—H19B	0.9900
C5—H5A	0.9900	C20—H20A	0.9900
C5—H5B	0.9900	C20—H20B	0.9900
C6—C7	1.524 (2)	O2—C21	1.244 (2)
C6—H6A	0.9900	O3—C21	1.287 (2)
C6—H6B	0.9900	O4—C25	1.362 (2)
C7—C8	1.517 (2)	O4—H4O	0.82 (2)
C7—H7A	0.9900	C21—C22	1.499 (2)
C7—H7B	0.9900	C22—C27	1.392 (2)
C8—H8A	0.9900	C22—C23	1.401 (2)
C8—H8B	0.9900	C23—C24	1.388 (2)
C9—C14	1.391 (2)	C23—H23	0.9500
C9—C10	1.393 (2)	C24—C25	1.392 (2)
C10—C11	1.389 (3)	C24—H24	0.9500
C10—H10	0.9500	C25—C26	1.400 (2)
C11—C12	1.383 (3)	C26—C27	1.383 (2)
C11—H11	0.9500	C26—H26	0.9500
C12—C13	1.384 (3)	C27—H27	0.9500
C12—H12	0.9500		
			
C1—O1—H1O	106.6 (15)	C13—C12—H12	120.3
C3—N1—C4	112.50 (13)	C12—C13—C14	119.97 (18)
C3—N1—C8	109.68 (13)	C12—C13—H13	120.0
C4—N1—C8	110.94 (13)	C14—C13—H13	120.0
C3—N1—H1N	109.6 (12)	C13—C14—C9	121.31 (16)
C4—N1—H1N	108.3 (13)	C13—C14—H14	119.3
C8—N1—H1N	105.6 (12)	C9—C14—H14	119.3
O1—C1—C9	106.91 (13)	C16—C15—C20	109.43 (14)
O1—C1—C2	107.21 (13)	C16—C15—C1	111.85 (14)
C9—C1—C2	111.47 (14)	C20—C15—C1	116.04 (15)
O1—C1—C15	107.90 (14)	C16—C15—H15	106.3
C9—C1—C15	112.59 (13)	C20—C15—H15	106.3
C2—C1—C15	110.48 (14)	C1—C15—H15	106.3
C3—C2—C1	111.46 (14)	C15—C16—C17	111.40 (15)
C3—C2—H2A	109.3	C15—C16—H16A	109.3
C1—C2—H2A	109.3	C17—C16—H16A	109.3
C3—C2—H2B	109.3	C15—C16—H16B	109.3
C1—C2—H2B	109.3	C17—C16—H16B	109.3
H2A—C2—H2B	108.0	H16A—C16—H16B	108.0
N1—C3—C2	112.92 (14)	C18—C17—C16	111.82 (17)
N1—C3—H3A	109.0	C18—C17—H17A	109.3
C2—C3—H3A	109.0	C16—C17—H17A	109.3
N1—C3—H3B	109.0	C18—C17—H17B	109.3
C2—C3—H3B	109.0	C16—C17—H17B	109.3
H3A—C3—H3B	107.8	H17A—C17—H17B	107.9
N1—C4—C5	110.01 (13)	C19—C18—C17	111.13 (14)
N1—C4—H4A	109.7	C19—C18—H18A	109.4
C5—C4—H4A	109.7	C17—C18—H18A	109.4
N1—C4—H4B	109.7	C19—C18—H18B	109.4
C5—C4—H4B	109.7	C17—C18—H18B	109.4
H4A—C4—H4B	108.2	H18A—C18—H18B	108.0
C4—C5—C6	111.40 (14)	C18—C19—C20	111.47 (16)
C4—C5—H5A	109.3	C18—C19—H19A	109.3
C6—C5—H5A	109.3	C20—C19—H19A	109.3
C4—C5—H5B	109.3	C18—C19—H19B	109.3
C6—C5—H5B	109.3	C20—C19—H19B	109.3
H5A—C5—H5B	108.0	H19A—C19—H19B	108.0
C7—C6—C5	110.52 (14)	C19—C20—C15	110.86 (15)
C7—C6—H6A	109.5	C19—C20—H20A	109.5
C5—C6—H6A	109.5	C15—C20—H20A	109.5
C7—C6—H6B	109.5	C19—C20—H20B	109.5
C5—C6—H6B	109.5	C15—C20—H20B	109.5
H6A—C6—H6B	108.1	H20A—C20—H20B	108.1
C8—C7—C6	111.06 (14)	C25—O4—H4O	107.3 (17)
C8—C7—H7A	109.4	O2—C21—O3	123.43 (16)
C6—C7—H7A	109.4	O2—C21—C22	119.08 (15)
C8—C7—H7B	109.4	O3—C21—C22	117.42 (14)
C6—C7—H7B	109.4	C27—C22—C23	118.22 (15)
H7A—C7—H7B	108.0	C27—C22—C21	119.29 (14)
N1—C8—C7	111.06 (14)	C23—C22—C21	122.17 (15)
N1—C8—H8A	109.4	C24—C23—C22	120.95 (15)
C7—C8—H8A	109.4	C24—C23—H23	119.5
N1—C8—H8B	109.4	C22—C23—H23	119.5
C7—C8—H8B	109.4	C23—C24—C25	119.81 (15)
H8A—C8—H8B	108.0	C23—C24—H24	120.1
C14—C9—C10	118.00 (16)	C25—C24—H24	120.1
C14—C9—C1	121.08 (15)	O4—C25—C24	118.21 (14)
C10—C9—C1	120.92 (16)	O4—C25—C26	121.89 (15)
C11—C10—C9	120.83 (17)	C24—C25—C26	119.90 (15)
C11—C10—H10	119.6	C27—C26—C25	119.45 (16)
C9—C10—H10	119.6	C27—C26—H26	120.3
C12—C11—C10	120.39 (17)	C25—C26—H26	120.3
C12—C11—H11	119.8	C26—C27—C22	121.58 (15)
C10—C11—H11	119.8	C26—C27—H27	119.2
C11—C12—C13	119.49 (18)	C22—C27—H27	119.2
C11—C12—H12	120.3		
			
O1—C1—C2—C3	−60.92 (18)	O1—C1—C15—C16	58.47 (19)
C9—C1—C2—C3	55.77 (18)	C9—C1—C15—C16	−59.3 (2)
C15—C1—C2—C3	−178.26 (14)	C2—C1—C15—C16	175.39 (15)
C4—N1—C3—C2	58.13 (19)	O1—C1—C15—C20	−175.03 (14)
C8—N1—C3—C2	−177.89 (14)	C9—C1—C15—C20	67.2 (2)
C1—C2—C3—N1	147.37 (15)	C2—C1—C15—C20	−58.1 (2)
C3—N1—C4—C5	−178.06 (14)	C20—C15—C16—C17	56.8 (2)
C8—N1—C4—C5	58.66 (18)	C1—C15—C16—C17	−173.14 (16)
N1—C4—C5—C6	−57.16 (18)	C15—C16—C17—C18	−55.2 (2)
C4—C5—C6—C7	54.9 (2)	C16—C17—C18—C19	53.4 (2)
C5—C6—C7—C8	−53.8 (2)	C17—C18—C19—C20	−54.7 (2)
C3—N1—C8—C7	176.68 (14)	C18—C19—C20—C15	57.6 (2)
C4—N1—C8—C7	−58.44 (18)	C16—C15—C20—C19	−58.0 (2)
C6—C7—C8—N1	55.9 (2)	C1—C15—C20—C19	174.29 (16)
O1—C1—C9—C14	167.46 (14)	O2—C21—C22—C27	−0.4 (2)
C2—C1—C9—C14	50.6 (2)	O3—C21—C22—C27	176.72 (15)
C15—C1—C9—C14	−74.2 (2)	O2—C21—C22—C23	−173.83 (16)
O1—C1—C9—C10	−13.2 (2)	O3—C21—C22—C23	3.3 (2)
C2—C1—C9—C10	−130.08 (16)	C27—C22—C23—C24	−2.1 (2)
C15—C1—C9—C10	105.11 (17)	C21—C22—C23—C24	171.35 (16)
C14—C9—C10—C11	0.1 (2)	C22—C23—C24—C25	−0.6 (3)
C1—C9—C10—C11	−179.21 (15)	C23—C24—C25—O4	−176.52 (15)
C9—C10—C11—C12	0.8 (3)	C23—C24—C25—C26	2.9 (3)
C10—C11—C12—C13	−1.0 (3)	O4—C25—C26—C27	176.85 (15)
C11—C12—C13—C14	0.4 (3)	C24—C25—C26—C27	−2.6 (2)
C12—C13—C14—C9	0.5 (3)	C25—C26—C27—C22	−0.2 (3)
C10—C9—C14—C13	−0.8 (2)	C23—C22—C27—C26	2.5 (3)
C1—C9—C14—C13	178.57 (15)	C21—C22—C27—C26	−171.18 (15)

### 1-(3-Cyclohexyl-3-hydroxy-3-phenylpropyl)piperidin-1-ium 4-hydroxybenzoate (II) Hydrogen-bond geometry (Å, º)

**Table:**

*D*—H···*A*	*D*—H	H···*A*	*D*···*A*	*D*—H···*A*
O1—H1*O*···O2	0.88 (3)	1.94 (3)	2.8068 (18)	165 (2)
N1—H1*N*···O3	0.94 (2)	1.76 (2)	2.6908 (19)	169.4 (17)
C2—H2*A*···O2	0.99	2.57	3.277 (2)	129
C4—H4*B*···O1^i^	0.99	2.40	3.278 (2)	148
C7—H7*A*···O3	0.99	2.64	3.314 (2)	126
O4—H4*O*···O3^ii^	0.82 (2)	1.84 (3)	2.6633 (18)	176 (3)
C26—H26···O3^ii^	0.95	2.62	3.281 (2)	127

Symmetry codes: (i) *x*, −*y*+1, *z*+1/2; (ii) *x*, −*y*, *z*−1/2.

### 1-(3-Cyclohexyl-3-hydroxy-3-phenylpropyl)piperidin-1-ium 4-bromobenzoate (III) Crystal data

**Table:**

C_20_H_32_NO^+^·C_7_H_4_BrO_2_^−^	*F*(000) = 1056
*M**_r_* = 502.48	*D*_x_ = 1.376 Mg m^−^^3^
Monoclinic, *P*2_1_/*n*	Mo *K*α radiation, λ = 0.71073 Å
*a* = 6.2422 (4) Å	Cell parameters from 9837 reflections
*b* = 17.8126 (14) Å	θ = 2.2–27.5°
*c* = 21.9938 (19) Å	µ = 1.72 mm^−^^1^
β = 97.345 (3)°	*T* = 90 K
*V* = 2425.4 (3) Å^3^	Rod, colourless
*Z* = 4	0.20 × 0.08 × 0.07 mm

### 1-(3-Cyclohexyl-3-hydroxy-3-phenylpropyl)piperidin-1-ium 4-bromobenzoate (III) Data collection

**Table:**

Bruker D8 Venture dual source diffractometer	5566 independent reflections
Radiation source: microsource	4634 reflections with *I* > 2σ(*I*)
Detector resolution: 7.41 pixels mm^-1^	*R*_int_ = 0.049
φ and ω scans	θ_max_ = 27.5°, θ_min_ = 1.9°
Absorption correction: multi-scan (*SADABS*; Krause *et al.*, 2015)	*h* = −8→8
*T*_min_ = 0.740, *T*_max_ = 0.862	*k* = −21→23
40844 measured reflections	*l* = −28→28

### 1-(3-Cyclohexyl-3-hydroxy-3-phenylpropyl)piperidin-1-ium 4-bromobenzoate (III) Refinement

**Table:**

Refinement on *F*^2^	Primary atom site location: structure-invariant direct methods
Least-squares matrix: full	Secondary atom site location: difference Fourier map
*R*[*F*^2^ > 2σ(*F*^2^)] = 0.037	Hydrogen site location: mixed
*wR*(*F*^2^) = 0.074	H atoms treated by a mixture of independent and constrained refinement
*S* = 1.13	*w* = 1/[σ^2^(*F*_o_^2^) + (0.0033*P*)^2^ + 2.553*P*] where *P* = (*F*_o_^2^ + 2*F*_c_^2^)/3
5566 reflections	(Δ/σ)_max_ = 0.001
334 parameters	Δρ_max_ = 0.33 e Å^−^^3^
68 restraints	Δρ_min_ = −0.38 e Å^−^^3^

### 1-(3-Cyclohexyl-3-hydroxy-3-phenylpropyl)piperidin-1-ium 4-bromobenzoate (III) Special details

**Table:**

Experimental. The crystal was mounted using polyisobutene oil on the tip of a fine glass fibre, which was fastened in a copper mounting pin with electrical solder. It was placed directly into the cold gas stream of a liquid-nitrogen based cryostat (Hope, 1994; Parkin & Hope, 1998). Diffraction data were collected with the crystal at 90K, which is standard practice in this laboratory for the majority of flash-cooled crystals.
Geometry. All esds (except the esd in the dihedral angle between two l.s. planes) are estimated using the full covariance matrix. The cell esds are taken into account individually in the estimation of esds in distances, angles and torsion angles; correlations between esds in cell parameters are only used when they are defined by crystal symmetry. An approximate (isotropic) treatment of cell esds is used for estimating esds involving l.s. planes.
Refinement. Refinement progress was checked using *Platon* (Spek, 2020) and by an *R*-tensor (Parkin, 2000). The final model was further checked with the IUCr utility *checkCIF*.

### 1-(3-Cyclohexyl-3-hydroxy-3-phenylpropyl)piperidin-1-ium 4-bromobenzoate (III) Fractional atomic coordinates and isotropic or equivalent isotropic displacement parameters (Å^2^)

**Table:**

	*x*	*y*	*z*	*U*_iso_*/*U*_eq_	Occ. (<1)
N1	−0.0077 (3)	0.73580 (11)	0.72464 (8)	0.0168 (4)	
H1N	0.057 (4)	0.6878 (14)	0.7274 (11)	0.020*	
O1	0.2444 (2)	0.71420 (9)	0.56823 (7)	0.0167 (3)	
H1O	0.277 (4)	0.6811 (15)	0.5928 (12)	0.025*	
C1	0.0138 (3)	0.71331 (12)	0.55325 (9)	0.0159 (4)	
C2	−0.0881 (3)	0.70260 (12)	0.61312 (9)	0.0158 (4)	
H2A	−0.247256	0.706091	0.604115	0.019*	
H2B	−0.051482	0.652058	0.630093	0.019*	
C3	−0.0063 (4)	0.76191 (13)	0.66025 (10)	0.0182 (5)	
H3A	0.142919	0.776098	0.654261	0.022*	
H3B	−0.097808	0.807276	0.653383	0.022*	
C4	−0.2313 (3)	0.72756 (13)	0.74133 (10)	0.0170 (5)	
H4A	−0.313965	0.692199	0.712543	0.020*	
H4B	−0.305374	0.776800	0.737427	0.020*	
C5	−0.2278 (4)	0.69889 (14)	0.80611 (10)	0.0229 (5)	
H5A	−0.165578	0.647694	0.809116	0.027*	
H5B	−0.377383	0.695961	0.816399	0.027*	
C6	−0.0946 (4)	0.75021 (18)	0.85167 (11)	0.0359 (7)	
H6A	−0.163886	0.800210	0.851709	0.043*	
H6B	−0.086857	0.728925	0.893473	0.043*	
C7	0.1317 (4)	0.7579 (2)	0.83370 (12)	0.0497 (9)	
H7A	0.216628	0.793139	0.862134	0.060*	
H7B	0.204808	0.708516	0.837223	0.060*	
C8	0.1239 (4)	0.78670 (18)	0.76852 (11)	0.0351 (7)	
H8A	0.060303	0.837693	0.765682	0.042*	
H8B	0.272461	0.790033	0.757489	0.042*	
C9	−0.0707 (13)	0.6559 (4)	0.5074 (3)	0.0136 (11)	0.508 (5)
C10	0.0709 (11)	0.6299 (3)	0.4673 (3)	0.0173 (14)	0.508 (5)
H10	0.213821	0.649190	0.470634	0.021*	0.508 (5)
C11	0.0044 (10)	0.5755 (4)	0.4219 (3)	0.0235 (14)	0.508 (5)
H11	0.101919	0.558842	0.394960	0.028*	0.508 (5)
C12	−0.2045 (12)	0.5462 (5)	0.4169 (4)	0.0273 (17)	0.508 (5)
H12	−0.249979	0.509381	0.386694	0.033*	0.508 (5)
C13	−0.3456 (12)	0.5716 (3)	0.4567 (3)	0.0207 (13)	0.508 (5)
H13	−0.487721	0.551647	0.453643	0.025*	0.508 (5)
C14	−0.2813 (14)	0.6259 (4)	0.5010 (3)	0.0136 (12)	0.508 (5)
H14	−0.380815	0.642943	0.527248	0.016*	0.508 (5)
C15	−0.039 (2)	0.7975 (6)	0.5267 (4)	0.0138 (11)	0.508 (5)
H15	0.015215	0.833325	0.560196	0.017*	0.508 (5)
C16	0.0816 (18)	0.8153 (6)	0.4728 (4)	0.0180 (14)	0.508 (5)
H16A	0.237713	0.805671	0.484359	0.022*	0.508 (5)
H16B	0.029602	0.781937	0.438020	0.022*	0.508 (5)
C17	0.049 (2)	0.8963 (7)	0.4531 (5)	0.0236 (9)	0.508 (5)
H17A	0.114029	0.929526	0.486503	0.028*	0.508 (5)
H17B	0.123234	0.905569	0.416712	0.028*	0.508 (5)
C18	−0.188 (2)	0.9152 (6)	0.4378 (4)	0.0220 (15)	0.508 (5)
H18A	−0.203745	0.969529	0.428832	0.026*	0.508 (5)
H18B	−0.248717	0.887262	0.400652	0.026*	0.508 (5)
C19	−0.313 (2)	0.8955 (8)	0.4901 (6)	0.0214 (18)	0.508 (5)
H19A	−0.468747	0.904270	0.477411	0.026*	0.508 (5)
H19B	−0.265974	0.928465	0.525560	0.026*	0.508 (5)
C20	−0.277 (2)	0.8140 (7)	0.5091 (6)	0.0169 (11)	0.508 (5)
H20A	−0.335805	0.780907	0.474794	0.020*	0.508 (5)
H20B	−0.356185	0.803143	0.544407	0.020*	0.508 (5)
C9'	−0.049 (2)	0.7826 (6)	0.5209 (3)	0.0138 (11)	0.492 (5)
C10'	0.1019 (19)	0.8232 (7)	0.4919 (4)	0.0180 (14)	0.492 (5)
H10'	0.245495	0.804915	0.493673	0.022*	0.492 (5)
C11'	0.045 (2)	0.8903 (7)	0.4604 (5)	0.0236 (9)	0.492 (5)
H11'	0.149052	0.916842	0.440915	0.028*	0.492 (5)
C12'	−0.166 (2)	0.9180 (6)	0.4577 (4)	0.0220 (15)	0.492 (5)
H12'	−0.204684	0.963546	0.436680	0.026*	0.492 (5)
C13'	−0.318 (2)	0.8781 (8)	0.4861 (6)	0.0214 (18)	0.492 (5)
H13'	−0.461470	0.896431	0.484072	0.026*	0.492 (5)
C14'	−0.261 (2)	0.8112 (8)	0.5175 (5)	0.0169 (11)	0.492 (5)
H14'	−0.365777	0.784779	0.536804	0.020*	0.492 (5)
C15'	−0.0302 (14)	0.6403 (5)	0.5099 (4)	0.0136 (11)	0.492 (5)
H15'	0.024398	0.595835	0.534938	0.016*	0.492 (5)
C16'	−0.2696 (15)	0.6269 (4)	0.4891 (4)	0.0173 (14)	0.492 (5)
H16C	−0.329759	0.669833	0.463868	0.021*	0.492 (5)
H16D	−0.347887	0.623333	0.525389	0.021*	0.492 (5)
C17'	−0.3021 (13)	0.5547 (4)	0.4518 (3)	0.0235 (14)	0.492 (5)
H17C	−0.257359	0.511378	0.478574	0.028*	0.492 (5)
H17D	−0.457395	0.548808	0.436556	0.028*	0.492 (5)
C18'	−0.1755 (13)	0.5547 (6)	0.3986 (4)	0.0273 (17)	0.492 (5)
H18C	−0.235017	0.593147	0.368608	0.033*	0.492 (5)
H18D	−0.190455	0.505129	0.378042	0.033*	0.492 (5)
C19'	0.0624 (11)	0.5707 (4)	0.4184 (4)	0.0207 (13)	0.492 (5)
H19C	0.127161	0.528577	0.443714	0.025*	0.492 (5)
H19D	0.138378	0.574622	0.381633	0.025*	0.492 (5)
C20'	0.0923 (11)	0.6428 (3)	0.4547 (3)	0.0136 (12)	0.492 (5)
H20C	0.040000	0.685599	0.428151	0.016*	0.492 (5)
H20D	0.247886	0.650653	0.468583	0.016*	0.492 (5)
Br1	1.04489 (4)	0.39057 (2)	0.85717 (2)	0.02363 (7)	
O2	0.1997 (3)	0.60868 (11)	0.73749 (8)	0.0373 (5)	
O3	0.3993 (2)	0.61835 (10)	0.66054 (7)	0.0260 (4)	
C21	0.3598 (3)	0.59223 (12)	0.71036 (11)	0.0194 (5)	
C22	0.5195 (3)	0.53769 (12)	0.74351 (10)	0.0167 (4)	
C23	0.4884 (3)	0.51112 (12)	0.80118 (10)	0.0179 (5)	
H23	0.359419	0.523381	0.817674	0.022*	
C24	0.6430 (3)	0.46708 (12)	0.8348 (1)	0.0194 (5)	
H24	0.622186	0.449348	0.874355	0.023*	
C25	0.8289 (3)	0.44938 (12)	0.80955 (10)	0.0177 (5)	
C26	0.8621 (4)	0.47302 (13)	0.75203 (10)	0.0203 (5)	
H26	0.989445	0.459348	0.735240	0.024*	
C27	0.7050 (4)	0.51743 (13)	0.71895 (10)	0.0198 (5)	
H27	0.724997	0.534080	0.679027	0.024*	

### 1-(3-Cyclohexyl-3-hydroxy-3-phenylpropyl)piperidin-1-ium 4-bromobenzoate (III) Atomic displacement parameters (Å^2^)

**Table:**

	*U*^11^	*U*^22^	*U*^33^	*U*^12^	*U*^13^	*U*^23^
N1	0.0140 (9)	0.0254 (11)	0.0109 (9)	−0.0013 (8)	0.0013 (7)	−0.0019 (8)
O1	0.0117 (7)	0.0229 (8)	0.0150 (8)	−0.0010 (6)	−0.0003 (6)	0.0023 (7)
C1	0.0122 (10)	0.0235 (12)	0.0118 (10)	−0.0025 (9)	0.0003 (8)	−0.0001 (9)
C2	0.0151 (10)	0.0199 (11)	0.0125 (10)	−0.0023 (8)	0.0023 (8)	0.0008 (9)
C3	0.0206 (11)	0.0214 (12)	0.0131 (11)	−0.0044 (9)	0.0039 (9)	−0.0015 (9)
C4	0.0121 (10)	0.0224 (12)	0.0166 (11)	−0.0006 (9)	0.0024 (8)	−0.0020 (9)
C5	0.0185 (11)	0.0355 (14)	0.0153 (11)	−0.0019 (10)	0.0050 (9)	−0.0003 (10)
C6	0.0314 (14)	0.066 (2)	0.0111 (12)	−0.0133 (13)	0.0044 (10)	−0.0086 (12)
C7	0.0289 (14)	0.106 (3)	0.0136 (13)	−0.0268 (16)	−0.0004 (11)	−0.0079 (15)
C8	0.0240 (13)	0.066 (2)	0.0152 (12)	−0.0213 (13)	0.0032 (10)	−0.0122 (13)
C9	0.010 (3)	0.016 (3)	0.0147 (13)	0.005 (2)	0.0003 (15)	−0.0024 (15)
C10	0.018 (2)	0.021 (2)	0.015 (3)	−0.0014 (17)	0.007 (2)	0.0028 (17)
C11	0.014 (3)	0.031 (2)	0.026 (3)	0.003 (2)	0.002 (2)	−0.0068 (19)
C12	0.031 (2)	0.018 (2)	0.033 (5)	−0.0048 (18)	0.005 (3)	−0.006 (3)
C13	0.012 (3)	0.027 (2)	0.023 (2)	0.0010 (19)	0.0031 (18)	−0.0044 (19)
C14	0.018 (2)	0.015 (2)	0.009 (2)	−0.0039 (16)	0.0064 (18)	0.0009 (16)
C15	0.0178 (16)	0.011 (4)	0.0123 (15)	−0.002 (2)	0.0011 (14)	−0.0014 (13)
C16	0.017 (2)	0.021 (2)	0.016 (5)	0.0001 (17)	0.002 (3)	0.002 (3)
C17	0.0258 (12)	0.0229 (19)	0.023 (2)	−0.0011 (13)	0.0057 (15)	0.0057 (17)
C18	0.031 (3)	0.0148 (14)	0.020 (5)	0.0005 (15)	0.000 (4)	−0.003 (3)
C19	0.0196 (12)	0.023 (6)	0.0208 (17)	0.002 (3)	0.0011 (12)	−0.003 (3)
C20	0.020 (2)	0.0185 (13)	0.013 (3)	−0.0013 (13)	0.0026 (19)	0.0011 (16)
C9'	0.0178 (16)	0.011 (4)	0.0123 (15)	−0.002 (2)	0.0011 (14)	−0.0014 (13)
C10'	0.017 (2)	0.021 (2)	0.016 (5)	0.0001 (17)	0.002 (3)	0.002 (3)
C11'	0.0258 (12)	0.0229 (19)	0.023 (2)	−0.0011 (13)	0.0057 (15)	0.0057 (17)
C12'	0.031 (3)	0.0148 (14)	0.020 (5)	0.0005 (15)	0.000 (4)	−0.003 (3)
C13'	0.0196 (12)	0.023 (6)	0.0208 (17)	0.002 (3)	0.0011 (12)	−0.003 (3)
C14'	0.020 (2)	0.0185 (13)	0.013 (3)	−0.0013 (13)	0.0026 (19)	0.0011 (16)
C15'	0.010 (3)	0.016 (3)	0.0147 (13)	0.005 (2)	0.0003 (15)	−0.0024 (15)
C16'	0.018 (2)	0.021 (2)	0.015 (3)	−0.0014 (17)	0.007 (2)	0.0028 (17)
C17'	0.014 (3)	0.031 (2)	0.026 (3)	0.003 (2)	0.002 (2)	−0.0068 (19)
C18'	0.031 (2)	0.018 (2)	0.033 (5)	−0.0048 (18)	0.005 (3)	−0.006 (3)
C19'	0.012 (3)	0.027 (2)	0.023 (2)	0.0010 (19)	0.0031 (18)	−0.0044 (19)
C20'	0.018 (2)	0.015 (2)	0.009 (2)	−0.0039 (16)	0.0064 (18)	0.0009 (16)
Br1	0.01870 (11)	0.02277 (12)	0.02920 (13)	0.00452 (10)	0.00219 (9)	0.00522 (11)
O2	0.0284 (9)	0.0486 (12)	0.0368 (11)	0.0198 (9)	0.0115 (8)	0.0151 (10)
O3	0.0226 (8)	0.0305 (10)	0.0236 (9)	0.0012 (7)	−0.0021 (7)	0.0081 (8)
C21	0.0183 (11)	0.0159 (11)	0.0225 (12)	−0.0012 (8)	−0.0035 (9)	−0.0023 (9)
C22	0.0182 (10)	0.0134 (10)	0.0179 (11)	−0.0007 (8)	−0.0003 (9)	−0.0033 (9)
C23	0.017 (1)	0.0150 (11)	0.0227 (12)	0.0003 (8)	0.0060 (9)	−0.0023 (9)
C24	0.0216 (11)	0.0171 (11)	0.0204 (12)	0.0005 (9)	0.0056 (9)	0.0011 (9)
C25	0.0179 (10)	0.0107 (10)	0.0237 (12)	0.0019 (8)	−0.0006 (9)	−0.0001 (9)
C26	0.0183 (11)	0.0197 (12)	0.0237 (12)	0.0027 (9)	0.0059 (9)	−0.0017 (10)
C27	0.0228 (11)	0.0203 (12)	0.0168 (11)	0.0009 (9)	0.0044 (9)	−0.0002 (9)

### 1-(3-Cyclohexyl-3-hydroxy-3-phenylpropyl)piperidin-1-ium 4-bromobenzoate (III) Geometric parameters (Å, º)

**Table:**

N1—C3	1.492 (3)	C18—C19	1.511 (8)
N1—C8	1.492 (3)	C18—H18A	0.9900
N1—C4	1.495 (3)	C18—H18B	0.9900
N1—H1N	0.95 (2)	C19—C20	1.519 (8)
O1—C1	1.436 (2)	C19—H19A	0.9900
O1—H1O	0.81 (3)	C19—H19B	0.9900
C1—C9'	1.454 (13)	C20—H20A	0.9900
C1—C9	1.485 (10)	C20—H20B	0.9900
C1—C2	1.546 (3)	C9'—C10'	1.404 (7)
C1—C15'	1.615 (11)	C9'—C14'	1.408 (7)
C1—C15	1.628 (13)	C10'—C11'	1.405 (7)
C2—C3	1.522 (3)	C10'—H10'	0.9500
C2—H2A	0.9900	C11'—C12'	1.396 (7)
C2—H2B	0.9900	C11'—H11'	0.9500
C3—H3A	0.9900	C12'—C13'	1.398 (7)
C3—H3B	0.9900	C12'—H12'	0.9500
C4—C5	1.511 (3)	C13'—C14'	1.401 (7)
C4—H4A	0.9900	C13'—H13'	0.9500
C4—H4B	0.9900	C14'—H14'	0.9500
C5—C6	1.522 (3)	C15'—C20'	1.516 (7)
C5—H5A	0.9900	C15'—C16'	1.525 (8)
C5—H5B	0.9900	C15'—H15'	1.0000
C6—C7	1.521 (4)	C16'—C17'	1.525 (7)
C6—H6A	0.9900	C16'—H16C	0.9900
C6—H6B	0.9900	C16'—H16D	0.9900
C7—C8	1.517 (4)	C17'—C18'	1.493 (7)
C7—H7A	0.9900	C17'—H17C	0.9900
C7—H7B	0.9900	C17'—H17D	0.9900
C8—H8A	0.9900	C18'—C19'	1.520 (7)
C8—H8B	0.9900	C18'—H18C	0.9900
C9—C10	1.405 (7)	C18'—H18D	0.9900
C9—C14	1.409 (7)	C19'—C20'	1.512 (7)
C10—C11	1.414 (6)	C19'—H19C	0.9900
C10—H10	0.9500	C19'—H19D	0.9900
C11—C12	1.396 (7)	C20'—H20C	0.9900
C11—H11	0.9500	C20'—H20D	0.9900
C12—C13	1.395 (7)	Br1—C25	1.911 (2)
C12—H12	0.9500	O2—C21	1.262 (3)
C13—C14	1.395 (6)	O3—C21	1.244 (3)
C13—H13	0.9500	C21—C22	1.511 (3)
C14—H14	0.9500	C22—C27	1.387 (3)
C15—C16	1.515 (8)	C22—C23	1.390 (3)
C15—C20	1.520 (8)	C23—C24	1.382 (3)
C15—H15	1.0000	C23—H23	0.9500
C16—C17	1.512 (8)	C24—C25	1.385 (3)
C16—H16A	0.9900	C24—H24	0.9500
C16—H16B	0.9900	C25—C26	1.374 (3)
C17—C18	1.511 (8)	C26—C27	1.391 (3)
C17—H17A	0.9900	C26—H26	0.9500
C17—H17B	0.9900	C27—H27	0.9500
			
C3—N1—C8	110.79 (18)	H17A—C17—H17B	107.9
C3—N1—C4	112.51 (16)	C19—C18—C17	111.3 (7)
C8—N1—C4	110.80 (18)	C19—C18—H18A	109.4
C3—N1—H1N	106.7 (15)	C17—C18—H18A	109.4
C8—N1—H1N	108.3 (14)	C19—C18—H18B	109.4
C4—N1—H1N	107.5 (15)	C17—C18—H18B	109.4
C1—O1—H1O	107.7 (18)	H18A—C18—H18B	108.0
O1—C1—C9'	107.6 (6)	C18—C19—C20	111.1 (7)
O1—C1—C9	115.0 (3)	C18—C19—H19A	109.4
O1—C1—C2	108.39 (16)	C20—C19—H19A	109.4
C9'—C1—C2	114.0 (5)	C18—C19—H19B	109.4
C9—C1—C2	110.2 (3)	C20—C19—H19B	109.4
O1—C1—C15'	103.5 (3)	H19A—C19—H19B	108.0
C9'—C1—C15'	112.3 (3)	C19—C20—C15	111.0 (7)
C2—C1—C15'	110.3 (3)	C19—C20—H20A	109.4
O1—C1—C15	102.8 (5)	C15—C20—H20A	109.4
C9—C1—C15	110.7 (3)	C19—C20—H20B	109.4
C2—C1—C15	109.5 (4)	C15—C20—H20B	109.4
C3—C2—C1	110.85 (17)	H20A—C20—H20B	108.0
C3—C2—H2A	109.5	C10'—C9'—C14'	118.0 (7)
C1—C2—H2A	109.5	C10'—C9'—C1	120.3 (11)
C3—C2—H2B	109.5	C14'—C9'—C1	121.7 (10)
C1—C2—H2B	109.5	C9'—C10'—C11'	121.3 (7)
H2A—C2—H2B	108.1	C9'—C10'—H10'	119.4
N1—C3—C2	112.83 (18)	C11'—C10'—H10'	119.4
N1—C3—H3A	109.0	C12'—C11'—C10'	120.0 (7)
C2—C3—H3A	109.0	C12'—C11'—H11'	120.0
N1—C3—H3B	109.0	C10'—C11'—H11'	120.0
C2—C3—H3B	109.0	C11'—C12'—C13'	119.4 (7)
H3A—C3—H3B	107.8	C11'—C12'—H12'	120.3
N1—C4—C5	111.28 (17)	C13'—C12'—H12'	120.3
N1—C4—H4A	109.4	C12'—C13'—C14'	120.6 (8)
C5—C4—H4A	109.4	C12'—C13'—H13'	119.7
N1—C4—H4B	109.4	C14'—C13'—H13'	119.7
C5—C4—H4B	109.4	C13'—C14'—C9'	120.8 (8)
H4A—C4—H4B	108.0	C13'—C14'—H14'	119.6
C4—C5—C6	111.0 (2)	C9'—C14'—H14'	119.6
C4—C5—H5A	109.4	C20'—C15'—C16'	109.9 (6)
C6—C5—H5A	109.4	C20'—C15'—C1	112.6 (6)
C4—C5—H5B	109.4	C16'—C15'—C1	112.9 (5)
C6—C5—H5B	109.4	C20'—C15'—H15'	107.0
H5A—C5—H5B	108.0	C16'—C15'—H15'	107.0
C7—C6—C5	109.3 (2)	C1—C15'—H15'	107.0
C7—C6—H6A	109.8	C15'—C16'—C17'	110.6 (6)
C5—C6—H6A	109.8	C15'—C16'—H16C	109.5
C7—C6—H6B	109.8	C17'—C16'—H16C	109.5
C5—C6—H6B	109.8	C15'—C16'—H16D	109.5
H6A—C6—H6B	108.3	C17'—C16'—H16D	109.5
C8—C7—C6	110.9 (2)	H16C—C16'—H16D	108.1
C8—C7—H7A	109.5	C18'—C17'—C16'	112.0 (6)
C6—C7—H7A	109.5	C18'—C17'—H17C	109.2
C8—C7—H7B	109.5	C16'—C17'—H17C	109.2
C6—C7—H7B	109.5	C18'—C17'—H17D	109.2
H7A—C7—H7B	108.0	C16'—C17'—H17D	109.2
N1—C8—C7	110.8 (2)	H17C—C17'—H17D	107.9
N1—C8—H8A	109.5	C17'—C18'—C19'	111.8 (6)
C7—C8—H8A	109.5	C17'—C18'—H18C	109.3
N1—C8—H8B	109.5	C19'—C18'—H18C	109.3
C7—C8—H8B	109.5	C17'—C18'—H18D	109.3
H8A—C8—H8B	108.1	C19'—C18'—H18D	109.3
C10—C9—C14	117.8 (6)	H18C—C18'—H18D	107.9
C10—C9—C1	117.2 (6)	C20'—C19'—C18'	111.2 (6)
C14—C9—C1	125.0 (5)	C20'—C19'—H19C	109.4
C9—C10—C11	121.2 (5)	C18'—C19'—H19C	109.4
C9—C10—H10	119.4	C20'—C19'—H19D	109.4
C11—C10—H10	119.4	C18'—C19'—H19D	109.4
C12—C11—C10	120.0 (5)	H19C—C19'—H19D	108.0
C12—C11—H11	120.0	C19'—C20'—C15'	111.0 (5)
C10—C11—H11	120.0	C19'—C20'—H20C	109.4
C13—C12—C11	119.1 (6)	C15'—C20'—H20C	109.4
C13—C12—H12	120.4	C19'—C20'—H20D	109.4
C11—C12—H12	120.4	C15'—C20'—H20D	109.4
C14—C13—C12	121.0 (6)	H20C—C20'—H20D	108.0
C14—C13—H13	119.5	O3—C21—O2	126.0 (2)
C12—C13—H13	119.5	O3—C21—C22	118.2 (2)
C13—C14—C9	120.9 (6)	O2—C21—C22	115.7 (2)
C13—C14—H14	119.5	C27—C22—C23	119.2 (2)
C9—C14—H14	119.5	C27—C22—C21	120.8 (2)
C16—C15—C20	109.2 (6)	C23—C22—C21	119.9 (2)
C16—C15—C1	112.1 (8)	C24—C23—C22	120.8 (2)
C20—C15—C1	114.5 (9)	C24—C23—H23	119.6
C16—C15—H15	106.9	C22—C23—H23	119.6
C20—C15—H15	106.9	C23—C24—C25	118.6 (2)
C1—C15—H15	106.9	C23—C24—H24	120.7
C17—C16—C15	111.1 (7)	C25—C24—H24	120.7
C17—C16—H16A	109.4	C26—C25—C24	122.1 (2)
C15—C16—H16A	109.4	C26—C25—Br1	119.58 (17)
C17—C16—H16B	109.4	C24—C25—Br1	118.28 (17)
C15—C16—H16B	109.4	C25—C26—C27	118.5 (2)
H16A—C16—H16B	108.0	C25—C26—H26	120.8
C18—C17—C16	111.7 (7)	C27—C26—H26	120.8
C18—C17—H17A	109.3	C22—C27—C26	120.8 (2)
C16—C17—H17A	109.3	C22—C27—H27	119.6
C18—C17—H17B	109.3	C26—C27—H27	119.6
C16—C17—H17B	109.3		
			
O1—C1—C2—C3	54.4 (2)	C1—C15—C20—C19	175.0 (6)
C9'—C1—C2—C3	−65.4 (5)	O1—C1—C9'—C10'	21.4 (4)
C9—C1—C2—C3	−179.0 (2)	C2—C1—C9'—C10'	141.6 (4)
C15'—C1—C2—C3	167.1 (3)	C15'—C1—C9'—C10'	−92.0 (5)
C15—C1—C2—C3	−57.0 (5)	O1—C1—C9'—C14'	−158.7 (5)
C8—N1—C3—C2	165.40 (19)	C2—C1—C9'—C14'	−38.4 (5)
C4—N1—C3—C2	−70.0 (2)	C15'—C1—C9'—C14'	88.0 (6)
C1—C2—C3—N1	−152.27 (17)	C14'—C9'—C10'—C11'	0.0 (4)
C3—N1—C4—C5	178.32 (18)	C1—C9'—C10'—C11'	180.0 (3)
C8—N1—C4—C5	−57.0 (3)	C9'—C10'—C11'—C12'	0.3 (6)
N1—C4—C5—C6	56.8 (3)	C10'—C11'—C12'—C13'	−0.5 (8)
C4—C5—C6—C7	−56.1 (3)	C11'—C12'—C13'—C14'	0.5 (8)
C5—C6—C7—C8	56.5 (4)	C12'—C13'—C14'—C9'	−0.3 (8)
C3—N1—C8—C7	−177.1 (2)	C10'—C9'—C14'—C13'	0.0 (6)
C4—N1—C8—C7	57.3 (3)	C1—C9'—C14'—C13'	−179.9 (4)
C6—C7—C8—N1	−57.7 (4)	O1—C1—C15'—C20'	−56.1 (5)
O1—C1—C9—C10	−23.8 (2)	C9'—C1—C15'—C20'	59.8 (8)
C2—C1—C9—C10	−146.6 (2)	C2—C1—C15'—C20'	−171.9 (4)
C15—C1—C9—C10	92.1 (6)	O1—C1—C15'—C16'	178.7 (4)
O1—C1—C9—C14	156.4 (3)	C9'—C1—C15'—C16'	−65.4 (8)
C2—C1—C9—C14	33.6 (3)	C2—C1—C15'—C16'	63.0 (6)
C15—C1—C9—C14	−87.7 (6)	C20'—C15'—C16'—C17'	56.9 (8)
C14—C9—C10—C11	0.0 (3)	C1—C15'—C16'—C17'	−176.4 (5)
C1—C9—C10—C11	−179.7 (2)	C15'—C16'—C17'—C18'	−55.4 (9)
C9—C10—C11—C12	−0.6 (5)	C16'—C17'—C18'—C19'	53.7 (9)
C10—C11—C12—C13	0.4 (7)	C17'—C18'—C19'—C20'	−54.1 (9)
C11—C12—C13—C14	0.4 (7)	C18'—C19'—C20'—C15'	56.5 (8)
C12—C13—C14—C9	−0.9 (7)	C16'—C15'—C20'—C19'	−58.1 (8)
C10—C9—C14—C13	0.7 (5)	C1—C15'—C20'—C19'	175.1 (5)
C1—C9—C14—C13	−179.5 (3)	O3—C21—C22—C27	0.0 (3)
O1—C1—C15—C16	57.2 (6)	O2—C21—C22—C27	−177.9 (2)
C9—C1—C15—C16	−66.0 (7)	O3—C21—C22—C23	175.7 (2)
C2—C1—C15—C16	172.3 (5)	O2—C21—C22—C23	−2.2 (3)
O1—C1—C15—C20	−177.7 (5)	C27—C22—C23—C24	2.1 (3)
C9—C1—C15—C20	59.1 (8)	C21—C22—C23—C24	−173.6 (2)
C2—C1—C15—C20	−62.6 (6)	C22—C23—C24—C25	−0.6 (3)
C20—C15—C16—C17	57.9 (9)	C23—C24—C25—C26	−1.2 (3)
C1—C15—C16—C17	−174.1 (7)	C23—C24—C25—Br1	178.47 (16)
C15—C16—C17—C18	−56.3 (9)	C24—C25—C26—C27	1.3 (3)
C16—C17—C18—C19	53.9 (9)	Br1—C25—C26—C27	−178.31 (17)
C17—C18—C19—C20	−54.1 (9)	C23—C22—C27—C26	−2.0 (3)
C18—C19—C20—C15	56.9 (9)	C21—C22—C27—C26	173.8 (2)
C16—C15—C20—C19	−58.3 (8)	C25—C26—C27—C22	0.3 (3)

### 1-(3-Cyclohexyl-3-hydroxy-3-phenylpropyl)piperidin-1-ium 4-bromobenzoate (III) Hydrogen-bond geometry (Å, º)

**Table:**

*D*—H···*A*	*D*—H	H···*A*	*D*···*A*	*D*—H···*A*
N1—H1*N*···O2	0.95 (2)	1.67 (3)	2.606 (3)	172 (2)
O1—H1*O*···O3	0.81 (3)	1.94 (3)	2.733 (2)	167 (3)
C3—H3*A*···Br1^i^	0.99	2.85	3.739 (2)	149
C4—H4*A*···O3^ii^	0.99	2.39	3.348 (3)	162

Symmetry codes: (i) −*x*+3/2, *y*+1/2, −*z*+3/2; (ii) *x*−1, *y*, *z*.

### 1-(3-Cyclohexyl-3-hydroxy-3-phenylpropyl)piperidin-1-ium thiophene-2-carboxylate hemihydrate (IV) Crystal data

**Table:**

2C_20_H_32_NO^+^·2C_5_H_3_O_2_S^−^·H_2_O	*Z* = 2
*M**_r_* = 877.21	*F*(000) = 948
Triclinic, *P*1	*D*_x_ = 1.226 Mg m^−^^3^
*a* = 6.2765 (3) Å	Mo *K*α radiation, λ = 0.71073 Å
*b* = 18.5390 (13) Å	Cell parameters from 9857 reflections
*c* = 20.6383 (14) Å	θ = 2.2–27.5°
α = 89.710 (2)°	µ = 0.16 mm^−^^1^
β = 81.600 (2)°	*T* = 90 K
γ = 88.977 (2)°	Cut block, colourless
*V* = 2375.3 (3) Å^3^	0.16 × 0.12 × 0.11 mm

### 1-(3-Cyclohexyl-3-hydroxy-3-phenylpropyl)piperidin-1-ium thiophene-2-carboxylate hemihydrate (IV) Data collection

**Table:**

Bruker D8 Venture dual source diffractometer	10922 independent reflections
Radiation source: microsource	8540 reflections with *I* > 2σ(*I*)
Detector resolution: 7.41 pixels mm^-1^	*R*_int_ = 0.049
φ and ω scans	θ_max_ = 27.5°, θ_min_ = 2.0°
Absorption correction: multi-scan (*SADABS*; Krause *et al.*, 2015)	*h* = −8→7
*T*_min_ = 0.908, *T*_max_ = 0.959	*k* = −24→24
78578 measured reflections	*l* = −26→26

### 1-(3-Cyclohexyl-3-hydroxy-3-phenylpropyl)piperidin-1-ium thiophene-2-carboxylate hemihydrate (IV) Refinement

**Table:**

Refinement on *F*^2^	Secondary atom site location: difference Fourier map
Least-squares matrix: full	Hydrogen site location: mixed
*R*[*F*^2^ > 2σ(*F*^2^)] = 0.042	H atoms treated by a mixture of independent and constrained refinement
*wR*(*F*^2^) = 0.101	*w* = 1/[σ^2^(*F*_o_^2^) + (0.0315*P*)^2^ + 1.4676*P*] where *P* = (*F*_o_^2^ + 2*F*_c_^2^)/3
*S* = 1.03	(Δ/σ)_max_ < 0.001
10922 reflections	Δρ_max_ = 0.71 e Å^−^^3^
603 parameters	Δρ_min_ = −0.30 e Å^−^^3^
168 restraints	Extinction correction: SHELXL-2019/2 (Sheldrick 2008), Fc^*^=kFc[1+0.001xFc^2^λ^3^/sin(2θ)]^-1/4^
Primary atom site location: structure-invariant direct methods	Extinction coefficient: 0.0025 (5)

### 1-(3-Cyclohexyl-3-hydroxy-3-phenylpropyl)piperidin-1-ium thiophene-2-carboxylate hemihydrate (IV) Special details

**Table:**

Experimental. The crystal was mounted using polyisobutene oil on the tip of a fine glass fibre, which was fastened in a copper mounting pin with electrical solder. It was placed directly into the cold gas stream of a liquid-nitrogen based cryostat (Hope, 1994; Parkin & Hope, 1998). Diffraction data were collected with the crystal at 90K, which is standard practice in this laboratory for the majority of flash-cooled crystals.
Geometry. All esds (except the esd in the dihedral angle between two l.s. planes) are estimated using the full covariance matrix. The cell esds are taken into account individually in the estimation of esds in distances, angles and torsion angles; correlations between esds in cell parameters are only used when they are defined by crystal symmetry. An approximate (isotropic) treatment of cell esds is used for estimating esds involving l.s. planes.
Refinement. Refinement progress was checked using *Platon* (Spek, 2020) and by an *R*-tensor (Parkin, 2000). The final model was further checked with the IUCr utility *checkCIF*.

### 1-(3-Cyclohexyl-3-hydroxy-3-phenylpropyl)piperidin-1-ium thiophene-2-carboxylate hemihydrate (IV) Fractional atomic coordinates and isotropic or equivalent isotropic displacement parameters (Å^2^)

**Table:**

	*x*	*y*	*z*	*U*_iso_*/*U*_eq_	Occ. (<1)
O1A	1.01018 (18)	0.31684 (6)	0.70649 (6)	0.0285 (3)	
H1OA	1.023 (3)	0.2634 (12)	0.7113 (10)	0.043*	
N1A	0.5203 (2)	0.23400 (7)	0.63156 (6)	0.0206 (3)	
H1NA	0.453 (3)	0.2036 (10)	0.6642 (9)	0.025*	
C1A	0.8139 (3)	0.34315 (8)	0.74407 (8)	0.0246 (3)	
C2A	0.6310 (3)	0.29422 (8)	0.73028 (8)	0.0253 (3)	
H2AA	0.675790	0.243307	0.735486	0.030*	
H2AB	0.502954	0.303999	0.763355	0.030*	
C3A	0.5673 (3)	0.30407 (8)	0.66211 (8)	0.0256 (3)	
H3AA	0.438076	0.335988	0.665222	0.031*	
H3AB	0.685613	0.328097	0.633470	0.031*	
C4A	0.3690 (3)	0.24531 (8)	0.58264 (8)	0.0239 (3)	
H4AA	0.433679	0.278891	0.548123	0.029*	
H4AB	0.233164	0.267659	0.604615	0.029*	
C5A	0.3196 (3)	0.17482 (9)	0.55142 (8)	0.0267 (3)	
H5AA	0.225973	0.184592	0.517602	0.032*	
H5AB	0.240637	0.143179	0.585165	0.032*	
C6A	0.5257 (3)	0.13633 (9)	0.52014 (8)	0.0301 (4)	
H6AA	0.490413	0.088829	0.503153	0.036*	
H6AB	0.596577	0.165251	0.482910	0.036*	
C7A	0.6776 (3)	0.12594 (9)	0.57060 (8)	0.0291 (4)	
H7AA	0.611910	0.092930	0.605468	0.035*	
H7AB	0.814144	0.103430	0.549390	0.035*	
C8A	0.7247 (2)	0.19724 (9)	0.60084 (8)	0.0249 (3)	
H8AA	0.819660	0.188632	0.634514	0.030*	
H8AB	0.801136	0.228876	0.566611	0.030*	
C9A	0.7819 (3)	0.42050 (8)	0.72132 (8)	0.0237 (3)	
C10A	0.9541 (3)	0.45935 (9)	0.68973 (8)	0.0273 (4)	
H10A	1.092921	0.437141	0.681494	0.033*	
C11A	0.9262 (3)	0.52997 (9)	0.67009 (8)	0.0321 (4)	
H11A	1.046247	0.555952	0.649098	0.038*	
C12A	0.7251 (3)	0.56292 (9)	0.68080 (9)	0.0326 (4)	
H12A	0.706126	0.611106	0.666536	0.039*	
C13A	0.5516 (3)	0.52542 (9)	0.71237 (9)	0.0309 (4)	
H13A	0.412744	0.547731	0.719791	0.037*	
C14A	0.5804 (3)	0.45499 (9)	0.73325 (8)	0.0280 (4)	
H14A	0.461284	0.429938	0.755983	0.034*	
C15A	0.8340 (3)	0.34294 (9)	0.81871 (8)	0.0266 (3)	
H15A	0.705784	0.370056	0.841580	0.032*	
C16A	0.8318 (3)	0.26799 (10)	0.84952 (9)	0.0354 (4)	
H16A	0.954228	0.238918	0.827028	0.042*	
H16B	0.697018	0.243715	0.843341	0.042*	
C17A	0.8478 (4)	0.27140 (12)	0.92268 (10)	0.0500 (6)	
H17A	0.718855	0.296857	0.945739	0.060*	
H17B	0.850162	0.221762	0.940516	0.060*	
C18A	1.0476 (4)	0.30993 (11)	0.9355 (1)	0.0495 (5)	
H18A	1.177279	0.282055	0.916199	0.059*	
H18B	1.048795	0.313333	0.983302	0.059*	
C19A	1.0534 (4)	0.38502 (11)	0.90591 (10)	0.0434 (5)	
H19A	1.190434	0.408055	0.911702	0.052*	
H19B	0.933691	0.414674	0.929198	0.052*	
C20A	1.0334 (3)	0.38268 (10)	0.83260 (9)	0.0333 (4)	
H20A	1.027564	0.432667	0.815760	0.040*	
H20B	1.163368	0.358562	0.808711	0.040*	
O1B	0.62051 (16)	0.22169 (6)	0.18905 (5)	0.0210 (2)	
H1OB	0.612 (3)	0.2411 (10)	0.2281 (10)	0.032*	
N1B	0.10474 (19)	0.12468 (7)	0.30623 (6)	0.0181 (3)	
H1NB	0.047 (3)	0.1674 (9)	0.3283 (8)	0.022*	
C1B	0.4188 (2)	0.23678 (8)	0.16663 (7)	0.0189 (3)	
C2B	0.2339 (2)	0.21738 (8)	0.22165 (7)	0.0193 (3)	
H2BA	0.216467	0.255748	0.255292	0.023*	
H2BB	0.097312	0.214315	0.203195	0.023*	
C3B	0.2815 (2)	0.14581 (8)	0.25303 (8)	0.0211 (3)	
H3BA	0.301071	0.107825	0.219030	0.025*	
H3BB	0.417797	0.149268	0.271543	0.025*	
C4B	−0.0739 (2)	0.08888 (9)	0.27877 (8)	0.0245 (3)	
H4BA	−0.015542	0.046408	0.252701	0.029*	
H4BB	−0.136153	0.122889	0.249186	0.029*	
C5B	−0.2491 (3)	0.06503 (9)	0.33256 (9)	0.0319 (4)	
H5BA	−0.359215	0.038520	0.312913	0.038*	
H5BB	−0.319904	0.108110	0.354948	0.038*	
C6B	−0.1609 (3)	0.01707 (10)	0.38237 (10)	0.0374 (4)	
H6BA	−0.277625	0.005040	0.418224	0.045*	
H6BB	−0.102964	−0.028468	0.361313	0.045*	
C7B	0.0161 (3)	0.05594 (10)	0.40982 (9)	0.0336 (4)	
H7BA	−0.044944	0.099505	0.433733	0.040*	
H7BB	0.077591	0.024010	0.441205	0.040*	
C8B	0.1921 (3)	0.07761 (9)	0.35551 (8)	0.0252 (3)	
H8BA	0.304572	0.103759	0.374417	0.030*	
H8BB	0.259651	0.033774	0.333591	0.030*	
C9B	0.4125 (2)	0.18947 (8)	0.10659 (7)	0.0201 (3)	
C10B	0.5955 (3)	0.15091 (9)	0.07854 (8)	0.0273 (4)	
H10B	0.723935	0.153051	0.097723	0.033*	
C11B	0.5920 (3)	0.10937 (10)	0.02280 (9)	0.0330 (4)	
H11B	0.717322	0.082911	0.004555	0.040*	
C12B	0.4076 (3)	0.10633 (10)	−0.00619 (8)	0.0321 (4)	
H12B	0.406621	0.078639	−0.044746	0.038*	
C13B	0.2245 (3)	0.14378 (9)	0.02120 (8)	0.0268 (4)	
H13B	0.097227	0.141749	0.001444	0.032*	
C14B	0.2258 (3)	0.18437 (9)	0.07746 (8)	0.0235 (3)	
H14B	0.098002	0.209034	0.096422	0.028*	
C15B	0.4105 (2)	0.31884 (8)	0.15073 (7)	0.0204 (3)	
H15B	0.439976	0.344282	0.191012	0.025*	
C16B	0.5900 (3)	0.34017 (9)	0.09634 (8)	0.0277 (4)	
H16C	0.565023	0.317958	0.054636	0.033*	
H16D	0.729811	0.321623	0.106890	0.033*	
C17B	0.5992 (3)	0.42217 (9)	0.08811 (9)	0.0321 (4)	
H17C	0.638612	0.443979	0.128317	0.039*	
H17D	0.712094	0.434314	0.051163	0.039*	
C18B	0.3841 (3)	0.4536 (1)	0.07513 (9)	0.0352 (4)	
H18C	0.353857	0.436755	0.032005	0.042*	
H18D	0.391733	0.506885	0.073601	0.042*	
C19B	0.2018 (3)	0.43126 (9)	0.12812 (9)	0.0294 (4)	
H19C	0.062788	0.449334	0.116573	0.035*	
H19D	0.222369	0.453327	0.170305	0.035*	
C20B	0.1949 (2)	0.34916 (9)	0.13579 (8)	0.0247 (3)	
H20C	0.079550	0.336296	0.171769	0.030*	
H20D	0.160938	0.327335	0.094916	0.030*	
O1C	0.38536 (19)	0.13539 (7)	0.72520 (6)	0.0311 (3)	
O2C	0.05586 (19)	0.17101 (6)	0.70831 (6)	0.0303 (3)	
C1C	0.1829 (3)	0.13457 (8)	0.73747 (8)	0.0232 (3)	
C2C	0.0836 (10)	0.0857 (2)	0.79077 (18)	0.0235 (3)	0.795 (2)
C3C	−0.1309 (7)	0.0756 (3)	0.8116 (3)	0.0320 (6)	0.795 (2)
H3C	−0.242434	0.101251	0.794336	0.038*	0.795 (2)
C4C	−0.1696 (6)	0.0221 (2)	0.86233 (17)	0.0270 (6)	0.795 (2)
H4C	−0.308005	0.005622	0.880602	0.032*	0.795 (2)
C5C	0.0185 (4)	−0.0016 (2)	0.88055 (17)	0.0257 (5)	0.795 (2)
H5C	0.026038	−0.034526	0.915589	0.031*	0.795 (2)
S1C	0.24428 (13)	0.03415 (5)	0.83360 (4)	0.0289 (2)	0.795 (2)
C2C'	0.092 (4)	0.0854 (8)	0.7908 (6)	0.0235 (3)	0.205 (2)
S1C'	−0.1819 (7)	0.0762 (3)	0.8081 (3)	0.0320 (6)	0.205 (2)
C4C'	−0.134 (3)	0.0206 (9)	0.8727 (7)	0.0270 (6)	0.205 (2)
H4C'	−0.248531	0.004734	0.904501	0.032*	0.205 (2)
C5C'	0.071 (2)	0.0010 (9)	0.8759 (7)	0.0257 (5)	0.205 (2)
H5C'	0.117994	−0.033274	0.905439	0.031*	0.205 (2)
C3C'	0.213 (2)	0.0421 (10)	0.8252 (8)	0.0289 (2)	0.205 (2)
H3C'	0.365342	0.038728	0.817909	0.035*	0.205 (2)
O1D	−0.07797 (17)	0.23654 (6)	0.37347 (5)	0.0263 (3)	
O2D	0.22275 (18)	0.26032 (7)	0.41459 (6)	0.0305 (3)	
C1D	0.0291 (2)	0.27138 (8)	0.41029 (7)	0.0210 (3)	
C2D	−0.0919 (3)	0.32908 (9)	0.45097 (9)	0.0194 (3)	0.953 (2)
C3D	−0.2973 (8)	0.3526 (4)	0.4525 (3)	0.0249 (7)	0.953 (2)
H3D	−0.393171	0.332453	0.426189	0.030*	0.953 (2)
C4D	−0.3574 (3)	0.40997 (11)	0.49666 (11)	0.0248 (4)	0.953 (2)
H4D	−0.496068	0.432443	0.503224	0.030*	0.953 (2)
C5D	−0.1924 (3)	0.42896 (11)	0.52857 (10)	0.0274 (4)	0.953 (2)
H5D	−0.201924	0.466275	0.560292	0.033*	0.953 (2)
S1D	0.03497 (9)	0.37763 (3)	0.50481 (3)	0.02668 (15)	0.953 (2)
C2D'	−0.065 (5)	0.3297 (10)	0.4521 (11)	0.0194 (3)	0.047 (2)
S1D'	−0.327 (5)	0.353 (2)	0.4487 (19)	0.0249 (7)	0.047 (2)
C4D'	−0.315 (6)	0.417 (2)	0.508 (2)	0.0248 (4)	0.047 (2)
H4D'	−0.433809	0.447017	0.525319	0.030*	0.047 (2)
C5D'	−0.118 (6)	0.421 (2)	0.526 (2)	0.0274 (4)	0.047 (2)
H5D'	−0.080477	0.454562	0.556402	0.033*	0.047 (2)
C3D'	0.027 (6)	0.369 (2)	0.494 (2)	0.02668 (15)	0.047 (2)
H3D'	0.172860	0.363239	0.500762	0.032*	0.047 (2)
O1W	0.5732 (3)	0.26595 (10)	0.31580 (7)	0.0497 (4)	
H1W1	0.679 (4)	0.2610 (13)	0.3359 (12)	0.059 (7)*	
H2W1	0.469 (4)	0.2601 (14)	0.3429 (13)	0.063 (8)*	

### 1-(3-Cyclohexyl-3-hydroxy-3-phenylpropyl)piperidin-1-ium thiophene-2-carboxylate hemihydrate (IV) Atomic displacement parameters (Å^2^)

**Table:**

	*U*^11^	*U*^22^	*U*^33^	*U*^12^	*U*^13^	*U*^23^
O1A	0.0283 (6)	0.0233 (6)	0.0313 (6)	0.0017 (5)	0.0045 (5)	0.0018 (5)
N1A	0.0209 (6)	0.0177 (6)	0.0221 (7)	−0.0006 (5)	0.0004 (5)	0.0043 (5)
C1A	0.0255 (8)	0.0209 (8)	0.0261 (8)	0.0008 (6)	0.0004 (6)	0.0029 (6)
C2A	0.0307 (9)	0.0189 (8)	0.0257 (8)	−0.0027 (6)	−0.0018 (7)	0.0040 (6)
C3A	0.0299 (8)	0.0163 (7)	0.0307 (9)	−0.0028 (6)	−0.0043 (7)	0.0032 (6)
C4A	0.0229 (8)	0.0241 (8)	0.0243 (8)	0.0020 (6)	−0.0023 (6)	0.0070 (6)
C5A	0.0275 (8)	0.0311 (9)	0.0215 (8)	−0.0028 (7)	−0.0039 (6)	0.0037 (7)
C6A	0.037 (1)	0.0282 (9)	0.0239 (8)	0.0000 (7)	0.0000 (7)	−0.0010 (7)
C7A	0.0325 (9)	0.0256 (9)	0.0274 (9)	0.0063 (7)	0.0015 (7)	0.0002 (7)
C8A	0.0200 (8)	0.0279 (8)	0.0255 (8)	0.0031 (6)	0.0007 (6)	0.0044 (7)
C9A	0.0304 (8)	0.0190 (8)	0.0228 (8)	−0.0005 (6)	−0.0071 (6)	−0.0002 (6)
C10A	0.0293 (9)	0.0258 (8)	0.0274 (8)	−0.0015 (7)	−0.0065 (7)	−0.0002 (7)
C11A	0.0437 (10)	0.0266 (9)	0.0269 (9)	−0.0098 (8)	−0.0076 (8)	0.0038 (7)
C12A	0.0499 (11)	0.0197 (8)	0.0306 (9)	−0.0013 (7)	−0.0142 (8)	0.0030 (7)
C13A	0.0369 (10)	0.0237 (9)	0.0337 (9)	0.0064 (7)	−0.0118 (8)	−0.0022 (7)
C14A	0.0306 (9)	0.0235 (8)	0.0301 (9)	−0.0022 (7)	−0.0046 (7)	0.0009 (7)
C15A	0.0307 (9)	0.0247 (8)	0.0232 (8)	−0.0011 (7)	−0.0006 (7)	0.0038 (6)
C16A	0.0495 (11)	0.0306 (9)	0.0255 (9)	−0.0034 (8)	−0.0034 (8)	0.0048 (7)
C17A	0.0773 (16)	0.0443 (12)	0.0286 (10)	−0.0043 (11)	−0.008 (1)	0.0103 (9)
C18A	0.0757 (16)	0.0418 (12)	0.0351 (11)	0.0015 (11)	−0.0222 (11)	0.0042 (9)
C19A	0.0621 (13)	0.0368 (11)	0.0354 (11)	0.0009 (9)	−0.0211 (10)	−0.0001 (8)
C20A	0.0373 (10)	0.0298 (9)	0.0343 (10)	−0.0011 (7)	−0.0108 (8)	0.0044 (7)
O1B	0.0169 (5)	0.0262 (6)	0.0203 (5)	0.0011 (4)	−0.0043 (4)	−0.0025 (4)
N1B	0.0163 (6)	0.0170 (6)	0.0206 (6)	−0.0003 (5)	−0.0013 (5)	0.0009 (5)
C1B	0.0153 (7)	0.0222 (8)	0.0194 (7)	0.0001 (6)	−0.0029 (6)	0.0010 (6)
C2B	0.0181 (7)	0.0203 (7)	0.0193 (7)	0.0000 (6)	−0.0019 (6)	0.0011 (6)
C3B	0.0171 (7)	0.0217 (8)	0.0230 (8)	−0.0001 (6)	0.0022 (6)	0.0023 (6)
C4B	0.0207 (8)	0.0236 (8)	0.0296 (9)	−0.0031 (6)	−0.0047 (6)	−0.0039 (7)
C5B	0.0203 (8)	0.0258 (9)	0.0474 (11)	−0.0043 (7)	0.0029 (7)	−0.0057 (8)
C6B	0.0362 (10)	0.0265 (9)	0.0429 (11)	−0.0036 (7)	0.0167 (8)	0.0047 (8)
C7B	0.038 (1)	0.0311 (9)	0.0284 (9)	0.0075 (8)	0.0051 (8)	0.0100 (7)
C8B	0.0230 (8)	0.0258 (8)	0.0262 (8)	0.0048 (6)	−0.0021 (6)	0.0061 (7)
C9B	0.0214 (7)	0.0203 (7)	0.0180 (7)	−0.0038 (6)	−0.0009 (6)	0.0012 (6)
C10B	0.0224 (8)	0.0339 (9)	0.0254 (8)	−0.0036 (7)	−0.0017 (6)	−0.0050 (7)
C11B	0.0287 (9)	0.0384 (10)	0.0304 (9)	−0.0012 (7)	0.0018 (7)	−0.0112 (8)
C12B	0.039 (1)	0.0349 (10)	0.0220 (8)	−0.0084 (8)	−0.0022 (7)	−0.0063 (7)
C13B	0.0307 (9)	0.0271 (9)	0.0246 (8)	−0.0078 (7)	−0.0094 (7)	0.0020 (7)
C14B	0.0241 (8)	0.0229 (8)	0.0239 (8)	−0.0026 (6)	−0.0044 (6)	0.0011 (6)
C15B	0.0198 (7)	0.0217 (8)	0.0201 (7)	−0.0021 (6)	−0.0035 (6)	0.0001 (6)
C16B	0.0242 (8)	0.0294 (9)	0.0285 (9)	−0.0031 (7)	−0.0005 (7)	0.0042 (7)
C17B	0.0304 (9)	0.0309 (9)	0.0343 (10)	−0.0084 (7)	−0.0017 (7)	0.0074 (7)
C18B	0.0392 (10)	0.0279 (9)	0.039 (1)	−0.0046 (8)	−0.0071 (8)	0.0099 (8)
C19B	0.0276 (9)	0.0258 (9)	0.0354 (9)	0.0012 (7)	−0.0071 (7)	0.0039 (7)
C20B	0.0230 (8)	0.0256 (8)	0.0258 (8)	−0.0018 (6)	−0.0046 (6)	0.0021 (6)
O1C	0.0290 (6)	0.0341 (7)	0.0305 (6)	−0.0095 (5)	−0.0043 (5)	0.0120 (5)
O2C	0.0370 (7)	0.0230 (6)	0.0301 (6)	0.0053 (5)	−0.0033 (5)	0.0039 (5)
C1C	0.0312 (8)	0.0177 (7)	0.0205 (7)	−0.0035 (6)	−0.0020 (6)	−0.0003 (6)
C2C	0.0316 (8)	0.0195 (7)	0.0189 (7)	−0.0046 (6)	−0.0018 (5)	−0.0006 (5)
C3C	0.0344 (15)	0.0275 (6)	0.0326 (8)	−0.0049 (11)	0.0006 (10)	−0.0001 (5)
C4C	0.0274 (13)	0.0286 (8)	0.0242 (12)	−0.0052 (8)	−0.0009 (8)	−0.0036 (9)
C5C	0.0285 (14)	0.0274 (9)	0.0204 (9)	−0.0103 (10)	−0.0005 (9)	0.0062 (7)
S1C	0.0347 (4)	0.0309 (4)	0.0241 (4)	−0.0118 (3)	−0.0131 (2)	0.0109 (2)
C2C'	0.0316 (8)	0.0195 (7)	0.0189 (7)	−0.0046 (6)	−0.0018 (5)	−0.0006 (5)
S1C'	0.0344 (15)	0.0275 (6)	0.0326 (8)	−0.0049 (11)	0.0006 (10)	−0.0001 (5)
C4C'	0.0274 (13)	0.0286 (8)	0.0242 (12)	−0.0052 (8)	−0.0009 (8)	−0.0036 (9)
C5C'	0.0285 (14)	0.0274 (9)	0.0204 (9)	−0.0103 (10)	−0.0005 (9)	0.0062 (7)
C3C'	0.0347 (4)	0.0309 (4)	0.0241 (4)	−0.0118 (3)	−0.0131 (2)	0.0109 (2)
O1D	0.0236 (6)	0.0275 (6)	0.0282 (6)	0.0051 (5)	−0.0051 (5)	−0.0084 (5)
O2D	0.0196 (6)	0.0374 (7)	0.0347 (7)	0.0048 (5)	−0.0053 (5)	0.0029 (5)
C1D	0.0210 (7)	0.0221 (8)	0.0194 (7)	−0.0005 (6)	−0.0017 (6)	0.0052 (6)
C2D	0.0225 (9)	0.0177 (7)	0.0182 (7)	−0.0036 (6)	−0.0029 (6)	0.0042 (6)
C3D	0.0237 (18)	0.0233 (8)	0.0274 (12)	−0.0019 (12)	−0.0026 (12)	−0.0010 (7)
C4D	0.0273 (10)	0.0185 (9)	0.0272 (11)	−0.0013 (7)	0.0013 (7)	−0.0007 (7)
C5D	0.0366 (12)	0.0192 (9)	0.0255 (9)	−0.0041 (8)	−0.0016 (9)	−0.0015 (7)
S1D	0.0310 (2)	0.0244 (3)	0.0271 (3)	−0.00499 (18)	−0.01186 (19)	−0.00035 (18)
C2D'	0.0225 (9)	0.0177 (7)	0.0182 (7)	−0.0036 (6)	−0.0029 (6)	0.0042 (6)
S1D'	0.0237 (18)	0.0233 (8)	0.0274 (12)	−0.0019 (12)	−0.0026 (12)	−0.0010 (7)
C4D'	0.0273 (10)	0.0185 (9)	0.0272 (11)	−0.0013 (7)	0.0013 (7)	−0.0007 (7)
C5D'	0.0366 (12)	0.0192 (9)	0.0255 (9)	−0.0041 (8)	−0.0016 (9)	−0.0015 (7)
C3D'	0.0310 (2)	0.0244 (3)	0.0271 (3)	−0.00499 (18)	−0.01186 (19)	−0.00035 (18)
O1W	0.0265 (7)	0.0958 (13)	0.0285 (7)	0.0131 (8)	−0.0105 (6)	−0.0212 (8)

### 1-(3-Cyclohexyl-3-hydroxy-3-phenylpropyl)piperidin-1-ium thiophene-2-carboxylate hemihydrate (IV) Geometric parameters (Å, º)

**Table:**

O1A—C1A	1.4356 (19)	C6B—H6BA	0.9900
O1A—H1OA	1.00 (2)	C6B—H6BB	0.9900
N1A—C4A	1.496 (2)	C7B—C8B	1.513 (2)
N1A—C8A	1.4989 (19)	C7B—H7BA	0.9900
N1A—C3A	1.499 (2)	C7B—H7BB	0.9900
N1A—H1NA	0.933 (18)	C8B—H8BA	0.9900
C1A—C9A	1.526 (2)	C8B—H8BB	0.9900
C1A—C2A	1.535 (2)	C9B—C10B	1.395 (2)
C1A—C15A	1.564 (2)	C9B—C14B	1.398 (2)
C2A—C3A	1.527 (2)	C10B—C11B	1.391 (2)
C2A—H2AA	0.9900	C10B—H10B	0.9500
C2A—H2AB	0.9900	C11B—C12B	1.381 (3)
C3A—H3AA	0.9900	C11B—H11B	0.9500
C3A—H3AB	0.9900	C12B—C13B	1.381 (2)
C4A—C5A	1.517 (2)	C12B—H12B	0.9500
C4A—H4AA	0.9900	C13B—C14B	1.388 (2)
C4A—H4AB	0.9900	C13B—H13B	0.9500
C5A—C6A	1.526 (2)	C14B—H14B	0.9500
C5A—H5AA	0.9900	C15B—C16B	1.526 (2)
C5A—H5AB	0.9900	C15B—C20B	1.528 (2)
C6A—C7A	1.520 (2)	C15B—H15B	1.0000
C6A—H6AA	0.9900	C16B—C17B	1.530 (2)
C6A—H6AB	0.9900	C16B—H16C	0.9900
C7A—C8A	1.517 (2)	C16B—H16D	0.9900
C7A—H7AA	0.9900	C17B—C18B	1.520 (3)
C7A—H7AB	0.9900	C17B—H17C	0.9900
C8A—H8AA	0.9900	C17B—H17D	0.9900
C8A—H8AB	0.9900	C18B—C19B	1.524 (2)
C9A—C10A	1.389 (2)	C18B—H18C	0.9900
C9A—C14A	1.396 (2)	C18B—H18D	0.9900
C10A—C11A	1.384 (2)	C19B—C20B	1.530 (2)
C10A—H10A	0.9500	C19B—H19C	0.9900
C11A—C12A	1.381 (3)	C19B—H19D	0.9900
C11A—H11A	0.9500	C20B—H20C	0.9900
C12A—C13A	1.382 (3)	C20B—H20D	0.9900
C12A—H12A	0.9500	O1C—C1C	1.2596 (19)
C13A—C14A	1.390 (2)	O2C—C1C	1.2533 (19)
C13A—H13A	0.9500	C1C—C2C'	1.482 (12)
C14A—H14A	0.9500	C1C—C2C	1.493 (4)
C15A—C16A	1.525 (2)	C2C—C3C	1.368 (7)
C15A—C20A	1.527 (2)	C2C—S1C	1.712 (6)
C15A—H15A	1.0000	C3C—C4C	1.436 (6)
C16A—C17A	1.529 (3)	C3C—H3C	0.9500
C16A—H16A	0.9900	C4C—C5C	1.356 (3)
C16A—H16B	0.9900	C4C—H4C	0.9500
C17A—C18A	1.512 (3)	C5C—S1C	1.735 (2)
C17A—H17A	0.9900	C5C—H5C	0.9500
C17A—H17B	0.9900	C2C'—C3C'	1.36 (2)
C18A—C19A	1.516 (3)	C2C'—S1C'	1.71 (2)
C18A—H18A	0.9900	S1C'—C4C'	1.738 (13)
C18A—H18B	0.9900	C4C'—C5C'	1.343 (12)
C19A—C20A	1.537 (3)	C4C'—H4C'	0.9500
C19A—H19A	0.9900	C5C'—C3C'	1.485 (15)
C19A—H19B	0.9900	C5C'—H5C'	0.9500
C20A—H20A	0.9900	C3C'—H3C'	0.9500
C20A—H20B	0.9900	O1D—C1D	1.2710 (19)
O1B—C1B	1.4327 (17)	O2D—C1D	1.2451 (18)
O1B—H1OB	0.88 (2)	C1D—C2D'	1.449 (17)
N1B—C8B	1.4947 (19)	C1D—C2D	1.490 (2)
N1B—C4B	1.4948 (19)	C2D—C3D	1.350 (4)
N1B—C3B	1.4981 (18)	C2D—S1D	1.724 (2)
N1B—H1NB	0.955 (18)	C3D—C4D	1.413 (5)
C1B—C9B	1.527 (2)	C3D—H3D	0.9500
C1B—C2B	1.546 (2)	C4D—C5D	1.358 (3)
C1B—C15B	1.556 (2)	C4D—H4D	0.9500
C2B—C3B	1.518 (2)	C5D—S1D	1.713 (2)
C2B—H2BA	0.9900	C5D—H5D	0.9500
C2B—H2BB	0.9900	C2D'—C3D'	1.331 (18)
C3B—H3BA	0.9900	C2D'—S1D'	1.706 (19)
C3B—H3BB	0.9900	S1D'—C4D'	1.715 (19)
C4B—C5B	1.514 (2)	C4D'—C5D'	1.347 (19)
C4B—H4BA	0.9900	C4D'—H4D'	0.9500
C4B—H4BB	0.9900	C5D'—C3D'	1.408 (19)
C5B—C6B	1.514 (3)	C5D'—H5D'	0.9500
C5B—H5BA	0.9900	C3D'—H3D'	0.9500
C5B—H5BB	0.9900	O1W—H1W1	0.84 (3)
C6B—C7B	1.513 (3)	O1W—H2W1	0.81 (3)
			
C1A—O1A—H1OA	110.2 (12)	C4B—C5B—H5BA	109.2
C4A—N1A—C8A	111.01 (12)	C6B—C5B—H5BA	109.2
C4A—N1A—C3A	110.95 (12)	C4B—C5B—H5BB	109.2
C8A—N1A—C3A	110.75 (12)	C6B—C5B—H5BB	109.2
C4A—N1A—H1NA	107.3 (11)	H5BA—C5B—H5BB	107.9
C8A—N1A—H1NA	107.9 (11)	C7B—C6B—C5B	109.36 (14)
C3A—N1A—H1NA	108.8 (11)	C7B—C6B—H6BA	109.8
O1A—C1A—C9A	106.32 (12)	C5B—C6B—H6BA	109.8
O1A—C1A—C2A	107.92 (13)	C7B—C6B—H6BB	109.8
C9A—C1A—C2A	111.94 (13)	C5B—C6B—H6BB	109.8
O1A—C1A—C15A	110.31 (13)	H6BA—C6B—H6BB	108.3
C9A—C1A—C15A	109.43 (13)	C8B—C7B—C6B	110.73 (15)
C2A—C1A—C15A	110.80 (13)	C8B—C7B—H7BA	109.5
C3A—C2A—C1A	114.04 (13)	C6B—C7B—H7BA	109.5
C3A—C2A—H2AA	108.7	C8B—C7B—H7BB	109.5
C1A—C2A—H2AA	108.7	C6B—C7B—H7BB	109.5
C3A—C2A—H2AB	108.7	H7BA—C7B—H7BB	108.1
C1A—C2A—H2AB	108.7	N1B—C8B—C7B	111.17 (13)
H2AA—C2A—H2AB	107.6	N1B—C8B—H8BA	109.4
N1A—C3A—C2A	112.66 (12)	C7B—C8B—H8BA	109.4
N1A—C3A—H3AA	109.1	N1B—C8B—H8BB	109.4
C2A—C3A—H3AA	109.1	C7B—C8B—H8BB	109.4
N1A—C3A—H3AB	109.1	H8BA—C8B—H8BB	108.0
C2A—C3A—H3AB	109.1	C10B—C9B—C14B	118.04 (14)
H3AA—C3A—H3AB	107.8	C10B—C9B—C1B	120.56 (14)
N1A—C4A—C5A	111.54 (13)	C14B—C9B—C1B	121.39 (13)
N1A—C4A—H4AA	109.3	C11B—C10B—C9B	120.71 (15)
C5A—C4A—H4AA	109.3	C11B—C10B—H10B	119.6
N1A—C4A—H4AB	109.3	C9B—C10B—H10B	119.6
C5A—C4A—H4AB	109.3	C12B—C11B—C10B	120.42 (16)
H4AA—C4A—H4AB	108.0	C12B—C11B—H11B	119.8
C4A—C5A—C6A	111.17 (14)	C10B—C11B—H11B	119.8
C4A—C5A—H5AA	109.4	C11B—C12B—C13B	119.61 (16)
C6A—C5A—H5AA	109.4	C11B—C12B—H12B	120.2
C4A—C5A—H5AB	109.4	C13B—C12B—H12B	120.2
C6A—C5A—H5AB	109.4	C12B—C13B—C14B	120.28 (15)
H5AA—C5A—H5AB	108.0	C12B—C13B—H13B	119.9
C7A—C6A—C5A	109.78 (14)	C14B—C13B—H13B	119.9
C7A—C6A—H6AA	109.7	C13B—C14B—C9B	120.91 (15)
C5A—C6A—H6AA	109.7	C13B—C14B—H14B	119.5
C7A—C6A—H6AB	109.7	C9B—C14B—H14B	119.5
C5A—C6A—H6AB	109.7	C16B—C15B—C20B	109.72 (13)
H6AA—C6A—H6AB	108.2	C16B—C15B—C1B	111.92 (12)
C8A—C7A—C6A	111.29 (14)	C20B—C15B—C1B	116.31 (12)
C8A—C7A—H7AA	109.4	C16B—C15B—H15B	106.0
C6A—C7A—H7AA	109.4	C20B—C15B—H15B	106.0
C8A—C7A—H7AB	109.4	C1B—C15B—H15B	106.0
C6A—C7A—H7AB	109.4	C15B—C16B—C17B	111.13 (14)
H7AA—C7A—H7AB	108.0	C15B—C16B—H16C	109.4
N1A—C8A—C7A	110.79 (13)	C17B—C16B—H16C	109.4
N1A—C8A—H8AA	109.5	C15B—C16B—H16D	109.4
C7A—C8A—H8AA	109.5	C17B—C16B—H16D	109.4
N1A—C8A—H8AB	109.5	H16C—C16B—H16D	108.0
C7A—C8A—H8AB	109.5	C18B—C17B—C16B	111.32 (14)
H8AA—C8A—H8AB	108.1	C18B—C17B—H17C	109.4
C10A—C9A—C14A	118.07 (15)	C16B—C17B—H17C	109.4
C10A—C9A—C1A	120.81 (14)	C18B—C17B—H17D	109.4
C14A—C9A—C1A	121.10 (14)	C16B—C17B—H17D	109.4
C11A—C10A—C9A	120.93 (16)	H17C—C17B—H17D	108.0
C11A—C10A—H10A	119.5	C17B—C18B—C19B	111.30 (14)
C9A—C10A—H10A	119.5	C17B—C18B—H18C	109.4
C12A—C11A—C10A	120.43 (17)	C19B—C18B—H18C	109.4
C12A—C11A—H11A	119.8	C17B—C18B—H18D	109.4
C10A—C11A—H11A	119.8	C19B—C18B—H18D	109.4
C11A—C12A—C13A	119.64 (16)	H18C—C18B—H18D	108.0
C11A—C12A—H12A	120.2	C18B—C19B—C20B	111.16 (14)
C13A—C12A—H12A	120.2	C18B—C19B—H19C	109.4
C12A—C13A—C14A	119.90 (16)	C20B—C19B—H19C	109.4
C12A—C13A—H13A	120.0	C18B—C19B—H19D	109.4
C14A—C13A—H13A	120.0	C20B—C19B—H19D	109.4
C13A—C14A—C9A	120.99 (16)	H19C—C19B—H19D	108.0
C13A—C14A—H14A	119.5	C15B—C20B—C19B	110.96 (13)
C9A—C14A—H14A	119.5	C15B—C20B—H20C	109.4
C16A—C15A—C20A	109.41 (15)	C19B—C20B—H20C	109.4
C16A—C15A—C1A	114.22 (14)	C15B—C20B—H20D	109.4
C20A—C15A—C1A	111.91 (13)	C19B—C20B—H20D	109.4
C16A—C15A—H15A	107.0	H20C—C20B—H20D	108.0
C20A—C15A—H15A	107.0	O2C—C1C—O1C	125.58 (15)
C1A—C15A—H15A	107.0	O2C—C1C—C2C'	118.5 (9)
C15A—C16A—C17A	111.76 (16)	O1C—C1C—C2C'	115.9 (9)
C15A—C16A—H16A	109.3	O2C—C1C—C2C	116.6 (3)
C17A—C16A—H16A	109.3	O1C—C1C—C2C	117.8 (3)
C15A—C16A—H16B	109.3	C3C—C2C—C1C	127.6 (5)
C17A—C16A—H16B	109.3	C3C—C2C—S1C	112.4 (3)
H16A—C16A—H16B	107.9	C1C—C2C—S1C	120.0 (4)
C18A—C17A—C16A	111.81 (18)	C2C—C3C—C4C	112.9 (5)
C18A—C17A—H17A	109.3	C2C—C3C—H3C	123.6
C16A—C17A—H17A	109.3	C4C—C3C—H3C	123.6
C18A—C17A—H17B	109.3	C5C—C4C—C3C	110.6 (4)
C16A—C17A—H17B	109.3	C5C—C4C—H4C	124.7
H17A—C17A—H17B	107.9	C3C—C4C—H4C	124.7
C17A—C18A—C19A	110.40 (18)	C4C—C5C—S1C	113.4 (3)
C17A—C18A—H18A	109.6	C4C—C5C—H5C	123.3
C19A—C18A—H18A	109.6	S1C—C5C—H5C	123.3
C17A—C18A—H18B	109.6	C2C—S1C—C5C	90.48 (19)
C19A—C18A—H18B	109.6	C3C'—C2C'—C1C	124.0 (16)
H18A—C18A—H18B	108.1	C3C'—C2C'—S1C'	116.2 (10)
C18A—C19A—C20A	111.35 (16)	C1C—C2C'—S1C'	119.6 (14)
C18A—C19A—H19A	109.4	C2C'—S1C'—C4C'	87.0 (8)
C20A—C19A—H19A	109.4	C5C'—C4C'—S1C'	117.5 (12)
C18A—C19A—H19B	109.4	C5C'—C4C'—H4C'	121.3
C20A—C19A—H19B	109.4	S1C'—C4C'—H4C'	121.3
H19A—C19A—H19B	108.0	C4C'—C5C'—C3C'	108.1 (13)
C15A—C20A—C19A	112.89 (16)	C4C'—C5C'—H5C'	125.9
C15A—C20A—H20A	109.0	C3C'—C5C'—H5C'	125.9
C19A—C20A—H20A	109.0	C2C'—C3C'—C5C'	110.3 (13)
C15A—C20A—H20B	109.0	C2C'—C3C'—H3C'	124.9
C19A—C20A—H20B	109.0	C5C'—C3C'—H3C'	124.9
H20A—C20A—H20B	107.8	O2D—C1D—O1D	124.94 (15)
C1B—O1B—H1OB	106.6 (12)	O2D—C1D—C2D'	112.5 (14)
C8B—N1B—C4B	111.36 (12)	O1D—C1D—C2D'	122.5 (14)
C8B—N1B—C3B	110.39 (11)	O2D—C1D—C2D	119.14 (15)
C4B—N1B—C3B	111.26 (12)	O1D—C1D—C2D	115.92 (14)
C8B—N1B—H1NB	107.5 (10)	C3D—C2D—C1D	129.6 (3)
C4B—N1B—H1NB	107.8 (10)	C3D—C2D—S1D	110.5 (2)
C3B—N1B—H1NB	108.4 (10)	C1D—C2D—S1D	119.83 (14)
O1B—C1B—C9B	106.73 (12)	C2D—C3D—C4D	114.2 (3)
O1B—C1B—C2B	108.88 (12)	C2D—C3D—H3D	122.9
C9B—C1B—C2B	110.47 (12)	C4D—C3D—H3D	122.9
O1B—C1B—C15B	107.46 (12)	C5D—C4D—C3D	111.6 (2)
C9B—C1B—C15B	112.84 (12)	C5D—C4D—H4D	124.2
C2B—C1B—C15B	110.27 (12)	C3D—C4D—H4D	124.2
C3B—C2B—C1B	110.51 (12)	C4D—C5D—S1D	111.97 (14)
C3B—C2B—H2BA	109.5	C4D—C5D—H5D	124.0
C1B—C2B—H2BA	109.5	S1D—C5D—H5D	124.0
C3B—C2B—H2BB	109.5	C5D—S1D—C2D	91.72 (9)
C1B—C2B—H2BB	109.5	C3D'—C2D'—C1D	128 (2)
H2BA—C2B—H2BB	108.1	C3D'—C2D'—S1D'	114.0 (15)
N1B—C3B—C2B	112.17 (12)	C1D—C2D'—S1D'	118 (2)
N1B—C3B—H3BA	109.2	C2D'—S1D'—C4D'	89.6 (13)
C2B—C3B—H3BA	109.2	C5D'—C4D'—S1D'	112.4 (18)
N1B—C3B—H3BB	109.2	C5D'—C4D'—H4D'	123.8
C2B—C3B—H3BB	109.2	S1D'—C4D'—H4D'	123.8
H3BA—C3B—H3BB	107.9	C4D'—C5D'—C3D'	113 (2)
N1B—C4B—C5B	111.40 (13)	C4D'—C5D'—H5D'	123.7
N1B—C4B—H4BA	109.3	C3D'—C5D'—H5D'	123.7
C5B—C4B—H4BA	109.3	C2D'—C3D'—C5D'	111.4 (19)
N1B—C4B—H4BB	109.3	C2D'—C3D'—H3D'	124.3
C5B—C4B—H4BB	109.3	C5D'—C3D'—H3D'	124.3
H4BA—C4B—H4BB	108.0	H1W1—O1W—H2W1	105 (2)
C4B—C5B—C6B	111.93 (14)		
			
O1A—C1A—C2A—C3A	−72.04 (17)	C9B—C10B—C11B—C12B	0.8 (3)
C9A—C1A—C2A—C3A	44.62 (18)	C10B—C11B—C12B—C13B	−1.2 (3)
C15A—C1A—C2A—C3A	167.08 (13)	C11B—C12B—C13B—C14B	0.1 (3)
C4A—N1A—C3A—C2A	154.13 (13)	C12B—C13B—C14B—C9B	1.4 (2)
C8A—N1A—C3A—C2A	−82.12 (16)	C10B—C9B—C14B—C13B	−1.8 (2)
C1A—C2A—C3A—N1A	140.13 (14)	C1B—C9B—C14B—C13B	177.10 (14)
C8A—N1A—C4A—C5A	56.52 (16)	O1B—C1B—C15B—C16B	61.74 (16)
C3A—N1A—C4A—C5A	−179.88 (12)	C9B—C1B—C15B—C16B	−55.65 (16)
N1A—C4A—C5A—C6A	−55.86 (17)	C2B—C1B—C15B—C16B	−179.72 (12)
C4A—C5A—C6A—C7A	54.99 (18)	O1B—C1B—C15B—C20B	−171.04 (12)
C5A—C6A—C7A—C8A	−55.89 (18)	C9B—C1B—C15B—C20B	71.57 (16)
C4A—N1A—C8A—C7A	−56.93 (16)	C2B—C1B—C15B—C20B	−52.50 (17)
C3A—N1A—C8A—C7A	179.36 (13)	C20B—C15B—C16B—C17B	57.42 (18)
C6A—C7A—C8A—N1A	57.21 (17)	C1B—C15B—C16B—C17B	−171.89 (13)
O1A—C1A—C9A—C10A	−20.6 (2)	C15B—C16B—C17B—C18B	−56.28 (19)
C2A—C1A—C9A—C10A	−138.19 (15)	C16B—C17B—C18B—C19B	54.5 (2)
C15A—C1A—C9A—C10A	98.57 (17)	C17B—C18B—C19B—C20B	−54.7 (2)
O1A—C1A—C9A—C14A	161.28 (15)	C16B—C15B—C20B—C19B	−57.63 (17)
C2A—C1A—C9A—C14A	43.7 (2)	C1B—C15B—C20B—C19B	174.07 (13)
C15A—C1A—C9A—C14A	−79.58 (19)	C18B—C19B—C20B—C15B	56.61 (18)
C14A—C9A—C10A—C11A	−0.6 (2)	O2C—C1C—C2C—C3C	0.3 (6)
C1A—C9A—C10A—C11A	−178.83 (15)	O1C—C1C—C2C—C3C	−179.1 (4)
C9A—C10A—C11A—C12A	−0.9 (3)	O2C—C1C—C2C—S1C	−179.70 (18)
C10A—C11A—C12A—C13A	1.1 (3)	O1C—C1C—C2C—S1C	0.9 (4)
C11A—C12A—C13A—C14A	0.1 (3)	C1C—C2C—C3C—C4C	177.5 (4)
C12A—C13A—C14A—C9A	−1.7 (3)	S1C—C2C—C3C—C4C	−2.5 (7)
C10A—C9A—C14A—C13A	1.9 (2)	C2C—C3C—C4C—C5C	4.5 (7)
C1A—C9A—C14A—C13A	−179.88 (15)	C3C—C4C—C5C—S1C	−4.6 (5)
O1A—C1A—C15A—C16A	−72.32 (18)	C3C—C2C—S1C—C5C	−0.1 (4)
C9A—C1A—C15A—C16A	171.04 (14)	C1C—C2C—S1C—C5C	179.9 (3)
C2A—C1A—C15A—C16A	47.13 (19)	C4C—C5C—S1C—C2C	2.8 (3)
O1A—C1A—C15A—C20A	52.69 (18)	O2C—C1C—C2C'—C3C'	178.7 (11)
C9A—C1A—C15A—C20A	−63.95 (17)	O1C—C1C—C2C'—C3C'	−0.8 (13)
C2A—C1A—C15A—C20A	172.14 (14)	O2C—C1C—C2C'—S1C'	3.4 (11)
C20A—C15A—C16A—C17A	54.7 (2)	O1C—C1C—C2C'—S1C'	−176.1 (6)
C1A—C15A—C16A—C17A	−178.95 (16)	C3C'—C2C'—S1C'—C4C'	8.2 (13)
C15A—C16A—C17A—C18A	−57.3 (2)	C1C—C2C'—S1C'—C4C'	−176.1 (10)
C16A—C17A—C18A—C19A	56.3 (2)	C2C'—S1C'—C4C'—C5C'	−9.2 (14)
C17A—C18A—C19A—C20A	−54.6 (2)	S1C'—C4C'—C5C'—C3C'	7.6 (18)
C16A—C15A—C20A—C19A	−54.0 (2)	C1C—C2C'—C3C'—C5C'	178.8 (12)
C1A—C15A—C20A—C19A	178.42 (14)	S1C'—C2C'—C3C'—C5C'	−5.7 (17)
C18A—C19A—C20A—C15A	54.8 (2)	C4C'—C5C'—C3C'—C2C'	−1.2 (18)
O1B—C1B—C2B—C3B	−43.39 (16)	O2D—C1D—C2D—C3D	176.8 (5)
C9B—C1B—C2B—C3B	73.51 (15)	O1D—C1D—C2D—C3D	−3.6 (5)
C15B—C1B—C2B—C3B	−161.07 (12)	O2D—C1D—C2D—S1D	−1.9 (2)
C8B—N1B—C3B—C2B	−152.81 (13)	O1D—C1D—C2D—S1D	177.75 (11)
C4B—N1B—C3B—C2B	83.03 (15)	C1D—C2D—C3D—C4D	−178.7 (3)
C1B—C2B—C3B—N1B	−179.42 (12)	S1D—C2D—C3D—C4D	0.0 (7)
C8B—N1B—C4B—C5B	54.34 (17)	C2D—C3D—C4D—C5D	−0.2 (7)
C3B—N1B—C4B—C5B	177.94 (13)	C3D—C4D—C5D—S1D	0.2 (4)
N1B—C4B—C5B—C6B	−54.87 (18)	C4D—C5D—S1D—C2D	−0.17 (17)
C4B—C5B—C6B—C7B	55.87 (19)	C3D—C2D—S1D—C5D	0.1 (4)
C5B—C6B—C7B—C8B	−57.11 (18)	C1D—C2D—S1D—C5D	178.98 (14)
C4B—N1B—C8B—C7B	−56.14 (17)	O2D—C1D—C2D'—C3D'	−2 (2)
C3B—N1B—C8B—C7B	179.76 (13)	O1D—C1D—C2D'—C3D'	179 (3)
C6B—C7B—C8B—N1B	57.95 (18)	O2D—C1D—C2D'—S1D'	177.5 (19)
O1B—C1B—C9B—C10B	−9.16 (19)	O1D—C1D—C2D'—S1D'	−2 (2)
C2B—C1B—C9B—C10B	−127.39 (15)	C3D'—C2D'—S1D'—C4D'	−2 (3)
C15B—C1B—C9B—C10B	108.65 (16)	C1D—C2D'—S1D'—C4D'	178.9 (19)
O1B—C1B—C9B—C14B	171.96 (13)	C2D'—S1D'—C4D'—C5D'	2 (4)
C2B—C1B—C9B—C14B	53.74 (18)	S1D'—C4D'—C5D'—C3D'	−2 (6)
C15B—C1B—C9B—C14B	−70.22 (18)	C1D—C2D'—C3D'—C5D'	−180 (3)
C14B—C9B—C10B—C11B	0.7 (2)	S1D'—C2D'—C3D'—C5D'	1 (3)
C1B—C9B—C10B—C11B	−178.20 (15)	C4D'—C5D'—C3D'—C2D'	1 (5)

### 1-(3-Cyclohexyl-3-hydroxy-3-phenylpropyl)piperidin-1-ium thiophene-2-carboxylate hemihydrate (IV) Hydrogen-bond geometry (Å, º)

**Table:**

*D*—H···*A*	*D*—H	H···*A*	*D*···*A*	*D*—H···*A*
O1*A*—H1*OA*···O2*C*^i^	1.00 (2)	1.72 (2)	2.7143 (16)	172.3 (18)
N1*A*—H1*NA*···O1*C*	0.933 (18)	1.793 (18)	2.7084 (17)	166.4 (16)
N1*A*—H1*NA*···O2*C*	0.933 (18)	2.606 (18)	3.3355 (18)	135.4 (14)
C4*A*—H4*AB*···O1*A*^ii^	0.99	2.51	3.407 (2)	150
C5*A*—H5*AA*···O2*D*	0.99	2.54	3.358 (2)	139
C8*A*—H8*AA*···O2*C*^i^	0.99	2.29	3.283 (2)	177
O1*B*—H1*OB*···O1*W*	0.88 (2)	1.85 (2)	2.7186 (18)	169.5 (18)
N1*B*—H1*NB*···O1*D*	0.955 (18)	1.700 (18)	2.6497 (17)	172.8 (16)
C3*B*—H3*BB*···O1*W*	0.99	2.61	3.295 (2)	126
O1*W*—H1*W*1···O1*D*^i^	0.84 (3)	1.86 (3)	2.6873 (18)	171 (2)
O1*W*—H2*W*1···O2*D*	0.81 (3)	1.98 (3)	2.775 (2)	171 (3)

Symmetry codes: (i) *x*+1, *y*, *z*; (ii) *x*−1, *y*, *z*.
